# Ammonifying and phosphorus-solubilizing function of *Aliikangiella maris* sp. nov. isolated from *Phaeocystis globosa* bloom and algal–bacterial interactions

**DOI:** 10.3389/fmicb.2025.1516993

**Published:** 2025-02-10

**Authors:** Fei Li, Ming-Ben Xu, Liang-Hao Pan, Jie Li, Cai-Bi Lan, Zhe Li, Shan Lu, Jun-Xiang Lai, Peng-Fu Li

**Affiliations:** ^1^State Key Laboratory of Pharmaceutical Biotechnology, School of Life Sciences, Nanjing University, Nanjing, China; ^2^Guangxi Key Laboratory of Marine Environmental Science, Guangxi Academy of Marine Sciences, Guangxi Academy of Sciences, Nanning, China; ^3^Guangxi Key Lab of Mangrove Conservation and Utilization, Guangxi Mangrove Research Center, Guangxi Academy of Marine Sciences, Beihai, China; ^4^Beibu Gulf Marine Industry Research Institute, Fangchenggang, China

**Keywords:** *Aliikangiella maris*, *Phaeocystis globosa* bloom, phycosphere bacterium, genome, algal–bacterial interactions

## Abstract

*Phaeocystis globosa* blooms are of escalating global concern due to their substantial ecological impacts on marine ecosystems. Emerging evidence indicates that algae–bacterial interactions play pivotal roles in shaping the ecology and evolution of harmful algal blooms, although much of this interplay remains unexplored. We successfully isolated and propagated two novel bacterial strains from *Phaeocystis globosa* bloom. Two novel Gram-negative, non-spore-forming, motile, rod-shaped, and yellow-pigmented bacteria were designated strains GXAS 306^T^ and GXAS 311. According to phenotypic, chemotaxonomic, phylogenomic, and comparative genomic analyses data, strains GXAS 306^T^ and GXAS 311 were considered to represent a novel species of the genus *Aliikangiella*. Genomic analysis revealed that strain GXAS 306^T^ had many potential functions favorable for interacting with algae, and further experimental evidence confirmed the ammonifying and phosphorus-solubilizing function. Co-culture experiments showed that strain GXAS 306^T^ significantly improved algal growth parameters of two typical *P. globosa* strains (Pg293 and PgV01), particularly under nitrogen or phosphorus deficiency. Specifically, cell densities were observed to increase by 19.6–86.0%, accompanied by substantial enhancements in photosynthetic performance with increases of 8.0–30.6% in *F*_v_*/F*_m_ and 10.9–27.9% in *r*_ETRmax_. Overall, these results shed light on intricate relationships between *P. globosa* and its associated bacterial partners, which may influence the growth characteristics of algae.

## Highlights

- A novel bacterium (GXAS 306^T^) was isolated from phycosphere in *P. globosa* bloom.
- GXAS 306^T^ had many potential functions in favor of interacting with algae.
- GXAS 306^T^ was demonstrated to have actual ammonifying and phosphorus-solubilizing function.
- GXAS 306^T^ positively regulated growth and physiological status of two typical *P. globosa* strains.

## 1 Introduction

*Phaeocystis globosa*, a key haptophyte species in marine ecosystems, exhibits a dual ecological role that significantly influences global biogeochemical cycles. As a prolific producer of dimethylsulfoniopropionate (DMSP), *P. globosa* plays a crucial role in climate regulation by facilitating the release of dimethylsulfide (DMS) into the atmosphere (Schoemann et al., 2005; Verity et al., 2007). However, this species also forms harmful algal blooms (HABs) that can cause damage such as massive death of oceanic life due to toxins and depletion of dissolved oxygen (Edvardsen and Imai, 2006; Nishibori et al., 2009) and formation of odorous foams on beaches (Schoemann et al., 2005).

*Phaeocystis globosa* emerges annually as a prominent bloom-forming species in Beibu Gulf (Su et al., 2022). Beibu Gulf is a natural semi-enclosed region of the South China Sea and suffers from extensive eutrophication due to nitrogen contamination (Han et al., 2012). The nitrogen-to-phosphorus ratios in most eutrophic sites are much higher than the Redfield value (16) in most years, indicating that water is in a phosphorus-limited state (Yang et al., 2015; Lu et al., 2022). When dissolved inorganic phosphorus pollution increases, eutrophication caused by nitrogen and phosphorus provides optimal conditions for the rampant propagation of *P. globosa*, resulting in the formation of blooms (Wang et al., 2011; Jiang et al., 2014; Wang et al., 2021; Chai et al., 2023). Moreover, eutrophication caused by nitrogen and phosphorus influences the progression and eventual demise of blooms. Scholarly consensus identifies the increase in dissolved inorganic nitrogen as a catalyst for blooms and the decrease in dissolved inorganic phosphorus as a bottleneck for blooms persistence (Qin et al., 2023; Xu M. et al., 2022).

The algae adapt to environmental changes by releasing organic matter to the surroundings, thus constructing a specialized microhabitat known as the “phycosphere,” facilitating interactions with neighboring organisms (Alderkamp et al., 2007; Mohamed, 2008). The bacterial community in the phycosphere participates in the formation, persistence, and termination of blooms through various mechanisms, including nutrient cycling, allelopathy, and signaling (Shao et al., 2014; Kouzuma and Watanabe, 2015). For instance, during the initial stages of *Microcystis aeruginosa* bloom, bacteria that are capable of nitrogen fixation may become more abundant, providing phytoplankton with a steady supply of nitrogen (Yang et al., 2017). Simultaneously, phycosphere bacteria engage in competition with phytoplankton for dissolved inorganic phosphorus (Xu S. et al., 2022). As the progression of *P. globosa* bloom and nutrient availability declines, specialized bacteria such as denitrifying bacteria and sulfate-reducing bacteria take center stage, optimizing nutrient recycling and salvaging (Shi et al., 2023). In particular, bacterial consumption of organic phosphate intensifies under such circumstances, leading to the increase in dissolved inorganic phosphorus (Xu S. et al., 2022). Such studies related to the genetic and metabolic exchanges between bacteria and *Phaeocystis* have been unveiled by omics technologies (Gibson et al., 2022; Xu S. et al., 2022; Shi et al., 2023). However, given the extensive functional redundancy among bacteria (Louca et al., 2018), the specifics of these interactions remain largely unexplored in *Phaeocystis* species, especially in individual bacterial species. Consequently, the importance of phycosphere bacteria must be considered for understanding the mechanisms of bloom formation in *P. globosa*.

In this study, two novel bacterial strains GXAS 306^T^ and GXAS 311 were isolated from *P. globosa* bloom in Beibu Gulf. A polyphasic taxonomic study revealed that strains GXAS 306^T^ and GXAS 311 represent a novel species of *Aliikangiella*, which is divided from the genus *Kangiella* by Wang et al. (2015). At the time of writing, the genus *Aliikangiella* contains two validly named species (List of Prokaryotic names with Standing in Nomenclature)^1^ with *A. marina* as the type species. The *Aliikangiella* belongs to the family Pleioneaceae (Wang et al., 2020), whose members are commonly related to the marine particulate organic matter degradation and appear as the central players in the marine nitrogen cycle (Pelve et al., 2007; Boeuf et al., 2019). Up to now, the study of interactions between Pleioneaceae and algae remains scant. *Kangiella* sp. N5, isolated from seawater, disrupts the chain structure of the bloom-forming alga *Skeletonema costatum* and shows algicidal activity (Shi et al., 2013). *Aliikangiella marina* is found to live in the culture broth of marine microalga *Picochlorum* sp. (Wang et al., 2015). In order to explore the interactions between *P. globosa* and phycosphere bacteria, the genomic characteristics and functions of strain GXAS 306^T^ related to algal–bacterial interactions were analyzed, and co-culture experiments were also devised.

## 2 Materials and methods

### 2.1 Polyphasic taxonomy study on strains GXAS 306^T^ and GXAS 311

#### 2.1.1 Isolation and maintenance of the microorganisms

Strains GXAS 306^T^ and GXAS 311 were isolated from surface seawater samples of *P. globosa* bloom in Qinzhou Bay (21°36′20”N, 108°34′51″E), Guangxi Zhuang Autonomous Region, China. The sampling time was selected during the massive algal bloom in January. The seawater sample was spread onto 2216E marine agar (MA, Hopebio, China) and Reasoner’s 2A agar (R2A, DIFCO, USA), respectively, and incubated at 30°C for 2 weeks. Colonies were selected and picked by morphology characteristics, such as color, shape, and transparency. Two yellow and transparent colonies, designated strains GXAS 306^T^ and GXAS 311, were all purified by streaking on MA plates and stored at −80°C in 20.0% (*v/v*) glycerol.

#### 2.1.2 Phenotypic characteristics

Strains GXAS 306^T^ and GXAS 311 were cultured using MA, R2A, International *Streptomyces* Project-2 agar (ISP2), tryptic soy agar (TSA, Solarbio, China), and nutrient agar (NA, DIFCO) at 30°C. Cellular morphology was observed using an optical microscope (Olympus BX53, Japan) and a transmission electron microscope (Thermo FEI Tecnai G2 spirit, USA) after incubation for 3 days in MA medium at 30°C. Gram staining was carried out by using a Gram-staining Kit (Solarbio) as described in the manufacturer’s instructions. Color evaluation was by comparing the cultures with the ISCC-NBS color charts (Kelly, 1964). NaCl tolerance for growth was performed using MA supplemented with 0–10% (*w/v*) NaCl (in 1% increments) at 30°C for 2 weeks. The temperature range for growth was examined on MA medium at 4, 10, 15, 20, 25, 30, 34, 37, and 40°C for 2 weeks. The pH range for growth was determined on MB medium (MA without agar) with pH 4.0–12.0 (at intervals of 0.5 pH unit and the buffer system described by Xu et al., 2005) at 30°C for 2 weeks. Catalase activity was determined by observing bubble production in a 3.0% (*v/v*) hydrogen peroxide solution, and oxidase activity was assessed using 1.0% (*w/v*) *N*, *N*, *N*′, *N*′-tetramethyl-1,4-phenylenediamine reagent (Sigma, USA). The peptonization of milk, production of H_2_S, and hydrolysis of casein, starch, Tween-20, Tween-60, and Tween-80 were examined as described previously (Gordon et al., 1974; Graham and Parker, 1964; Yokota et al., 1993). Metabolic capabilities were tested using API 20NE and API ZYM strips. Anaerobic fermentation was determined using the API 50CH strips with API 50 CHB medium according to the manufacturer’s protocol (Wang et al., 2015).

#### 2.1.3 Phylogenetic and genome sequencing analysis

Phylogenetic analysis of strains GXAS 306^T^ and GXAS 311 was performed based on 16S rRNA gene sequences. Genomic DNA of the two strains were isolated from pure cultures using “Chelex 100” chelating resin (De Lamballerie et al., 1992). The 16S rRNA gene of strains was amplified from genomic DNA by PCR using Ex Taq PCR premix (Sangon, China) and the universal bacterial primer pair 27F and 1492R (Li et al., 2007). PCR products were cloned into the pEASY-T1 Cloning Kit (Takara, Japan) and sequenced at the Sangon Biotech (Shanghai) Co., Ltd. Alignment of 16S rRNA gene sequences was compared with that of type strains in EzBioCloud Service (Yoon et al., 2017a,b) and performed using SINA 1.2.12 software package (Pruesse et al., 2012) in the silva rRNA database. Phylogenetic trees were reconstructed using the maximum-likelihood (Felsenstein, 1981), neighbor-joining (Saitou and Nei, 1987), and maximum-parsimony (Swofford, 1993) algorithms in software package mega version 7.0 (Kumar et al., 2016). The phylogenetic distance matrices were estimated by the Kimura two-parameter model (Kimura, 1980). The topology of the phylogenetic tree was evaluated by using the bootstrap resampling method by Felsenstein (1985) with 1,000 replicates.

Genomic DNA of strains GXAS 306^T^ and GXAS 311 was extracted using a Bacterial Genomic DNA Isolation Kit (Sangon) for sequencing. Whole-genome sequencing was performed using an Illumina HiSeq PE150 platform. Library construction was performed by PCR amplification of a 400-bp insert with A-tail ligated to full-length adaptors at Shanghai Major Biomedical Technology Co., Ltd. All good-quality paired reads were assembled using SOAPdenovo^2^ (Li et al., 2008; Li et al., 2010) into a number of scaffolds. Genome information was extracted according to Chun et al. (2018). The draft genome sequences of strains GXAS 306^T^ and GXAS 311 have been deposited at DDBJ/ENA/GenBank. To further explore the phylogenetic position of strains GXAS 306^T^ and GXAS 311, some housekeeping genes in their genome were aligned using autoMLST^3^ (Alanjary et al., 2019). Moreover, the sequence alignment results reconstructed the maximum-likelihood phylogenetic tree according to the previously mentioned method. The average nucleotide identity (ANI) and average amino acid identity (AAI) values were analyzed on the online tool of Majorbio Cloud Platform^4^ (Ren et al., 2022). The digital DNA–DNA hybridization (dDDH) was calculated using the genome-to-genome distance calculator^5^ (Meier-Kolthoff et al., 2013), respectively. Additionally, the automated genome annotation was carried out using the Rapid Annotation using Subsystem Technology (RAST) (Aziz et al., 2008), Kyoto Encyclopedia of Genes and Genomes (KEGG) (Kanehisa et al., 2004), and Carbohydrate-Active Enzymes database (CAZy) (Cantarel et al., 2009).

#### 2.1.4 Chemotaxonomic characterization

For cellular fatty acid analysis, the biomass of strains GXAS 306^T^, GXAS 311, and reference strain was acquired from the third quadrant of the quadrant streaked MA plate incubated at 30°C and then collected in the late-exponential stage. Cells were subjected to saponification, methylation, and extraction (Sasser, 1990). In addition, cellular fatty acid composition was analyzed by gas chromatography (Agilent G6890N, USA). For respiratory quinone and polar lipids analyses, strains GXAS 306^T^, GXAS 311, and reference strain were cultured in a 2.0-L Erlenmeyer flask containing 600 mL of MB in a rotary shaker (180 revolutions per minute, rpm) at 30°C for 3 days, respectively. Respiratory quinones were extracted as described by Collins (1994) and analyzed using reversed-phase high-performance liquid chromatography (Komagata and Suzuki, 1987). The polar lipids were extracted and analyzed by two-dimensional thin-layer chromatography on silica gel 60 F254 plates (Merck) as described by Minnikin et al. (1984). The solvent systems of the first and the second dimensions were chloroform–methanol–water (64:27:5, *v/v/v*) and chloroform–acetic acid–methanol–water (80:18:12:5, *v/v/v/v*), respectively.

### 2.2 Utilization of nitrogen and phosphorus

#### 2.2.1 Abilities of degrading organic nitrogen

To evaluate the proficiency of strain GXAS 306^T^ in metabolizing organic nitrogen sources, particularly in conjunction with inorganic nitrogen compounds such as nitrate and ammonium, a series of experiments were designed using a revised MB medium (peptone, 5.0 g; yeast extract, 1.0 g; ferric citrate, 0.1 g; NaCl, 20.0 g; MgCl_2_, 6.0 g; Na_2_SO_4_, 3.0 g; CaCl_2_, 2.0 g; K_2_HPO_4_, 0.2 g; distilled water, 1,000 mL). A revised MB medium was employed as the baseline to test the capability of strain GXAS 306^T^ in organic nitrogen degradation. Subsequently, to examine how inorganic nitrogen impacted organic nitrogen utilization of strain GXAS 306^T^, 0.1 g/L NaNO_3_ or (NH_4_)_2_SO_4_ was added to the revised MB medium. The bacterial suspension was then inoculated into the above-designed medium. For negative control, bacterial suspension was replaced by an equal amount of sterile physiological saline (0.7%, *w/v*). The preparation method for bacterial suspension was described as follows. The strain was cultured in a 500 mL Erlenmeyer flask containing 200 mL of MB medium in a rotary shaker at 180 rpm and 30°C. The bacterial culture in the exponential phase was collected by centrifugation at 10000 × *g*, washed three times, and resuspended in sterile physiological saline. The bacterial biomass was measured every 24 h by plate counting method. The concentration of total nitrogen (TN), nitrate nitrogen (NO_3_^−^-N), and ammoniacal nitrogen (NH_4_^+^-N) in the culture medium was measured by a segmented continuous flow analyzer (Skalar SAN++, Holland).

#### 2.2.2 Abilities of dissolving unavailable phosphorus

To evaluate the capability of strain GXAS 306^T^ to solubilize insoluble phosphorus (both inorganic and organic phosphorus), a qualitative screening was performed. The strain was streaked onto a phosphorus-solubilizing agar medium (Hopebio) and incubated at 30°C for 7 days. After the incubation period, the plates were inspected for bacterial growth and the formation of transparent circles (halos) around the colonies. Subsequently, a more detailed quantitative analysis was conducted to measure the actual extent of phosphorus solubilization by strain GXAS 306^T^. The bacterial suspension was inoculated into the phosphorus-solubilizing medium (without agar, Hopebio) at 30°C on a rotary shaker at 180 rpm. For negative control, bacterial suspension was replaced by an equal amount of sterile physiological saline. The preparation for bacterial suspension was the same as above. The bacterial biomass was measured every 24 h by plate counting method. The concentration of the soluble phosphorus (PO_4_^3−^-P) in the medium was determined and calculated according to the method described by Zhang et al. (2016).

### 2.3 Co-culture of *Phaeocystis globosa* and strain GXAS 306^T^

#### 2.3.1 Bacterial utilization of algal extracellular products

To investigate the effects of *P. globosa* extracellular products on the growth of strain GXAS 306^T^, a series of steps were carefully executed. The strain was inoculated into a 250-mL Erlenmeyer flask containing 100 mL of sterile filtrate of *P. globosa* in a rotary shaker at 180 rpm and 30°C. The sterile filtrate of *P. globosa* was prepared as follows. *P. globosa* culture of late-exponential phase (approximately 2.5 L) was centrifuged to collect the supernatant. The supernatant filtered with a 0.22-μm sterilized syringe filter was inoculated into a 250-mL sterile Erlenmeyer flask. The optical density at 600 nm of bacterial culture was measured initially and after 7-day incubation to determine bacterial growth. The dissolved organic carbon (DOC) content of the filtrate was determined with a TOC analyzer (Elementor, Vario TOC cube, Germany).

#### 2.3.2 Impact of strain GXAS 306^T^ on the growth of *Phaeocystis globosa*

To understand the interactions of *P. globosa* with strain GXAS 306^T^, we performed the following test. *P. globosa* 293 (Pg293) from Beibu Gulf and *P. globosa* V01 (PgV01) from Vietnam were employed in this study and supplied by the Culture Collection of Marine Algae, Guangxi Academy of Science. They were maintained in Erlenmeyer flask with f/10 medium (NaNO_3_, 0.015 g; NaH_2_PO_4_, 0.00113 g; f/2 trace metal solution, 0.2 mL; f/2 vitamin solution, 0.1 mL; distilled water, 1,000 mL; no silicate was added; f/2 medium according to Guillard, 1975) under 60 μmol photons m^−2^ s^−1^ irradiance with a 12 h:12 h light: dark cycle. The salinity of the medium was 25 psu, and the culture temperature was 20°C. Algal suspension was centrifuged at 3500 × *g* for 5 min to pellet the cells, and the washing procedure was repeated three times to ensure the removal of dissolved nitrogen and phosphorus. After the final wash, the algal cells were resuspended in artificial seawater and cultured for 12 h. Subsequently, the cell density of that was adjusted to approximately 10^5^ cells/mL. Meanwhile, strain GXAS 306^T^ was collected at the exponential stage, and then, the washing procedure was repeated three times to ensure the removal of the residual medium. After the final wash, the biomass of strain GXAS 306^T^ was resuspended in artificial physiological saline. The bacterial suspension was introduced into the algal culture, and the biomass was adjusted at a final concentration of 10^5^ CFU/mL. The pure culture of PgV01 and Pg293 served as the control. These organisms were co-cultured in f/10 medium to preliminarily understand the relationship between strain GXAS 306^T^ and *P. globosa*.

To further investigate the influence of strain GXAS 306^T^ on the growth of *P. globosa* under nitrogen and phosphorus deficiency, the f/10 medium was revised to exclude specific nutrients (NaH_2_PO_4_ or NaNO_3_). The pure culture of PgV01 and Pg293 served as the control, which was cultured in the revised medium as described above. The growth of *P. globosa* was measured every 24 h by direct counting of cells using a 100 μL counting plate with an optical microscope (Olympus BX53). Additionally, primary photochemistry (*F*_v_*/F*_m_) and maximum relative electron transfer rate (*r*_ETRmax_) were measured with a phytoplankton analyzer (WALZ, Rohrdorf, Germany) according to Zhang et al. (2015). All samples were exposed to a dark environment for 6 min before measurement. All physiological indicators were measured in triplicate and reported as the mean standard deviation.

### 2.4 Statistical analysis

Two-way ANOVA and least significant difference (LSD) tests for multiple comparisons were performed to evaluate the differences in cell counts and chlorophyll fluorescence index of *P. globosa* among available P and/or N treatments. All of the analyses were performed using statistical program SPSS 16.0, with a significance level of 5%.

## 3 Results and discussion

### 3.1 Taxonomic study of strains GXAS 306^T^ and GXAS 311

#### 3.1.1 Phenotypic characteristics

Cells of strains GXAS 306^T^ and GXAS 311 were Gram-negative, non-spore-forming, aerobic, short rods, and motile with single polar flagellum (Supplementary Figures S1A,B). They grew well on MA and R2A, but poorly on TSA and NA. Cells could grow with 0–4% NaCl (*w/v*), at pH 5.5–10.5 and 15–37°C. Optimal growth occurred with 2% NaCl (*w/v*) at pH 7 and 28–30°C. Compared with the type strains of *Aliikangiella*, the isolates were less NaCl halotolerant in terms of growth. In addition, cells were positive for catalase, casein, Tween-60 and Tween-80 hydrolyzation, and peptonization of milk. Production of H_2_S, oxidase activity, and hydrolysis of cellulose and starch were negative. In API 50CH tests, acid was produced from D-arabinose, D-ribose, L-sorbose, myo-inositol, D-mannitol, methyl *α*-D-mannopyranoside, methyl α-D-glucopyranoside, N-acetyl-D-glucosamine, arbutin, starch, D-maltose, D-melezitose, glycogen, xylitol, turanose, L-fucose, and 5-ketogluconate. In API ZYM tests, strains GXAS 306^T^ and GXAS 311 were positive for alkaline phosphatase, esterase (C4), esterase lipase (C8), leucine arylamidase, valine arylamidase, cystine arylamidase, trypsin, α-chymotrypsin, and naphthol AS-BI-phosphohydrolase. The other phenotypic characteristics of strains GXAS 306^T^ and GXAS 311 by API 50CH, API 20NE, and API ZYM tests were negative. Strains GXAS 306^T^ and GXAS 311 could be distinguished by some basic characteristics from closely related reference strains, namely *A. marina* GYP-15^T^, *A. coralliicola* M105^T,^ and *Pleionea sediminis* S1-5-21^T^. Moreover, those physiological and biochemical characteristics were summarized and compared in Table 1.

**Table 1:** Major characteristics that distinguished strain BGMRC 0090^T^ from the closest recognized species.

Characteristics	1	2	3^||^	4^†^	5^§^
Cell size (width×length, μm)	0.2–0.8 × 1.2–2.8	ND	0.2–0.4 × 2.6–4.3^*^	0.2–0.4 × 1.3–3.6	1.7–4.0 × 0.5–1.0
Growth pH range	5.5–10.5	5.5–10.0	6.0–9.0 (7.0–8.0)	7–8	5–8 (7)
Growth temperature (°C)	15–37 (28–30)	15–37 (28–30)	15–37 (30)	15–40 (25–30)	15–40 (30–35)
NaCl concentration for growth (%, w/v)	0–4 (2–3)	0–5 (3)	2–8 (2–3)	0–10 (1–3)	0.5–10 (1)
Oxidase	−	−	+	−	−
Tween-20	+	+	+	−	ND
Tween-80	+	+	−	−	−
Peptonization of milk	+	+	−	ND	−
Enzyme activities (API ZYM)					
Lipase (C14)	−	−	w	−	w
Leucine arylamidase	+	w	−	+	+
Acid phosphatase	−	−	+	+	+
Naphthol AS-BI-phosphohydrolase	w	+	−	+	+
API 50 CH					
D-arabinose	w	+	−	−	ND
D-ribose	w	+	+	−	ND
L-sorbose	+	w	−	−	ND
Myo-inositol	+	w	−	−	ND
D-mannitol	+	+	−	−	ND
D-maltose	w	+	−	−	ND
D-melezitose	w	+	−	−	ND
Polar lipids	PE, PG, DPG, GL, APL, AL, L	PE, PG, DPG, AL, L	PE, PG, DPG, APL, L	PE, PG, DPG, NL	PE, PG, DPG, AL, PL, L

Strains: 1, GXAS 306^T^; 2, GXAS 311; 3, *Aliikangiella marina* GYP-15^T^; 4, *Aliikangiella coralliicola* M105^T^; 5, *Pleionea sediminis* S1-5-21^T^. +, Positive; −, negative; w, weakly positive; ND, no data available. DPG, diphosphatidylglycerol; PE, phosphatidylethanolamine; PG, phosphatidylglycerol; GL, unidentified glycolipid (s); AL, unknown aminolipid (s); PL, unidentified phospholipid (s); APL, unidentified aminophospholipid (s); NL, unidentified ninhydrin-positive lipid; L, unidentified lipid (s).

* Data from Wang et al. (2015).

† Data from Wang et al. (2020).

§ Data from Luo et al. (2019).

|| Results different from those reported in the original descriptions.

#### 3.1.2 Phylogenetic analysis

**Figure 1:**
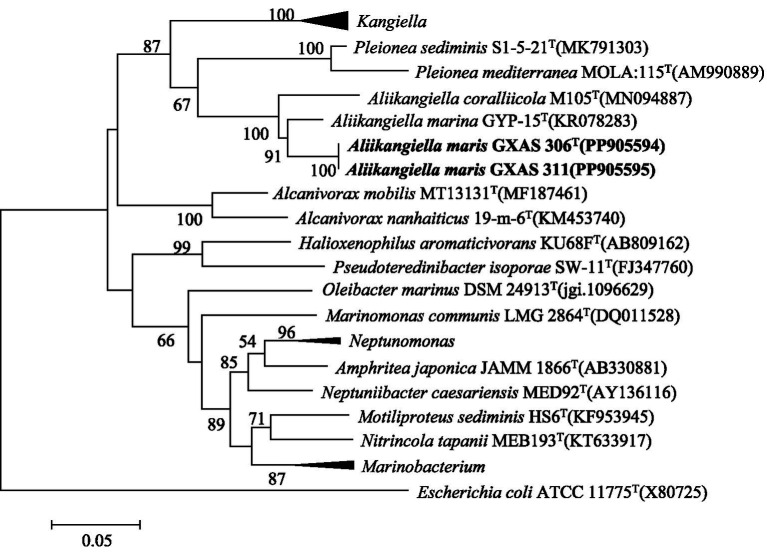
Maximum-likelihood phylogenetic tree based on 16S rRNA gene sequences showing the relationships among strain GXAS 306^T^, GXAS 311, and closely related taxa. Scale bar indicates 0.05 substitutions per nucleotide position. *Escherichia coli* ATCC 11775^T^ (X80725) was used as outgroup. GenBank accession numbers for each sequence are listed in parentheses.

Sequence identity calculation indicated that strain GXAS 306^T^ (GenBank accession number PP905594) and GXAS 311 (GenBank accession number PP905595) belong to the family Pleioneaceae in the order Oceanospirillales. Pairwise comparison of 16S rRNA gene sequences showed that strains GXAS 306^T^ and GXAS 311 had the highest similarity with *A. marina* GYP-15^T^ (95.8%), followed by *A*. *coralliicola* M105^T^ (94.2%), *P. sediminis* S1-5-21^T^ (90.1%), and *K. profundi* FT102^T^ (90.1%). Based on the 16S rRNA gene sequences phylogenetic trees using the maximum-likelihood algorithms, strains GXAS 306^T^ and GXAS 311 were located within the genus *Aliikangiella* but formed a separated clade with the related species (Figure 1). The topologies of strains GXAS 306^T^ and GXAS 311 cluster were similar and stable in the maximum-likelihood, neighbor-joining, and maximum-parsimony trees (Supplementary Figures S2, S3).

#### 3.1.3 Genomic characteristics

The Illumina sequencing results indicated the genome sequence length of strains GXAS 306^T^ and GXAS 311 was approximately 5,379,662 bp and 5,049,449 bp, respectively. The whole genome G + C content was 38.5 mol%. For reference strain GYP-15^T^, a total of 5,336,331bp genome sequences were obtained from eight scaffolds, and the whole genome G + C content was 41.9 mol%. The general genomic features of strains GXAS 306^T^ and GXAS 311 and their phylogenetic neighbors are listed in Supplementary Table S1. The ANI, AAI, and dDDH values of strain GXAS 306^T^ and seven close species were 67.2–69.9%, 56.4–67.0%, and 17.9–24.1% (Supplementary Table S2), which were lower than the threshold values of ANI (95 ~ 96%), AAI (95.0 ~ 95.5%), and DDH (70%) to discriminate bacterial species (Li F. et al., 2023), respectively, while the ANI, AAI, and dDDH values of strains GXAS 306^T^ and GXAS 311 were all above 99.8%. According to the recommendations of Wayne (1988), this value permits the classification of the two isolates as members of the same species. Moreover, the phylogenomic trees based on 83 housekeeping genes showed that strains GXAS 306^T^ and GXAS 311 formed an independent branch with *A. marina* GYP-15^T^ and *A*. *coralliicola* M105^T^ with high bootstrap values at nodes (Supplementary Figure S4), generally consistent with the phylogenetic trees based on 16S rRNA gene sequences.

Bacterial gene function distribution in strains GXAS 306^T^ and GXAS 311 was applied based on genome-scale metabolic reconstruction using the RAST annotation engine. Strains GXAS 306^T^ and GXAS 311 contained a large number of genes related to amino acids and derivatives (357 and 290), protein metabolism (271 and 187), cofactors, vitamins, prosthetic groups, and pigments (236 and 151), fatty acids, lipids and isoprenoids (164 and 66), RNA metabolism (163 and 46), carbohydrates (160 and 88), stress response (157 and 72), membrane transport (98 and 80), DNA metabolism (95 and 64), and respiration (92 and 69). Compared to other members, strains GXAS 306^T^ and GXAS 311 shared 83.0–85.1% of functions compared with their neighbors and had 30 unique functions (Supplementary Table S3). More genomic characteristics and gene annotation of strain GXAS 306^T^ are analyzed by the KEGG database. The annotation results showed that strain GXAS 306^T^ has 13 genes related to the selenocompound metabolism, including assimilation, methylation, and reduction in selenium, indicating that the strain can tolerate high concentrations of SeO_4_^2−^ or SeO_3_^2−^ to protect itself or symbiote from poisons. In particular, strain GXAS 306^T^ had a complete biosynthesis pathway (PPD) for heme, as a cofactor binding protein in organisms, playing a role in electron transfer, gas transport, chemical catalysis, metabolism, and detoxification.

Genomic characteristics related to algal–bacterial interactions were observed based on the KEGG analysis. Strain GXAS 306^T^ has many genes or gene clusters in favor of coexisting with algae, which is related to phosphorus solubilizing, nitrate assimilation, and ammonia assimilation (Supplementary Table S4), the synthesis of plant growth hormones and various vitamins (Supplementary Table S5), and LysR-type transcriptional regulator (LTTRs) (Supplementary Table S6).

#### 3.1.4 Chemotaxonomic characterization

The cellular fatty acid profile of strains GXAS 306^T^ and GXAS 311 was different from that of *A. marina* GYP-15^T^, as shown in Table 2. Major cellular fatty acids (>5%) of strains GXAS 306^T^ and GXAS 311 were iso-C_15:0_, summed feature 9, C_14:0_ DMA, C_18:0,_ and C_16:0_. Among them, iso-C_15:0_ and summed feature 9 were characteristic in the fatty acid compositions of *Aliikangiella* species (Wang et al., 2015). However, the two novel isolates could be distinguished from related species by significant differences in iso-C_16:0_, which was one of the main fatty acids (>5%) in *A. marina* GYP-15^T^, as described by Wang et al. (2015). Strain GXAS 306^T^, GXAS 311, and *A. marina* GYP-15^T^ had the same respiratory quinone (ubiquinone 8). Major polar lipid components of strains GXAS 306^T^ and GXAS 311 were diphosphatidylglycerol (DPG), phosphatidylethanolamine (PE), and phosphatidylglycerol (PG), which were classic profile in *Aliikangiella* species. Strain GXAS 306^T^ has more unknown polar lipids such as three unknown aminolipids (AL), two unknown glycolipids (GL), one unidentified amino phospholipid (APL), and one unknown lipid (L) (Supplementary Figure S5).

**Table 2:** Cellular fatty acid composition (%) of strain GXAS 306^T^, GXAS 311, and *A. marina* GYP-15^T^.

Fatty acids	1	2	3
C_12:0_	3.89	4.05	3.15
C_14:0_	–	–	1.07
C_14:0_ DMA	**10.03**	**10.14**	**4.29**
C_16:0_	**7.55**	**6.62**	**4.69**
C_18:0_	**6.98**	**7.84**	**2.35**
Iso–C_13:0_	1.70	1.41	1.64
Iso–C_14:0_	4.78	2.65	5.70
Iso–C_15:0_	**14.70**	**15.42**	**16.71**
Iso–C_16:0_	–	–	6.14
Iso–C_17:0_	1.20	–	1.86
Iso–C_18:0_	4.68	4.70	4.32
Iso–C_19:0_	3.39	4.55	3.18
anteiso C_14:0_	2.76	–	3.09
anteiso C_15:0_	1.27	1.15	1.19
anteiso C_16:0_	1.80	1.35	1.67
anteiso C_17:0_	2.63	1.84	2.37
C_14:1_ ω5c	2.76	3.96	2.91
Summed feature 3	4.44	3.52	4.73
Summed feature 5	1.91	1.94	1.83
Summed feature 8	–	2.66	2.17
Summed feature 9	**13.23**	**10.40**	**14.32**

Strains: 1, GXAS 306^T^; 2, GXAS 311; 3, *A. marina* GYP-15^T^. For fatty acid analysis, all strains were cultured on MA at 30°C. All data were from this study. –, less than 1% of total fatty acids. Bold font, the main fatty acids.

Summed features consist of two or more fatty acids that cannot be separated by the MIDI system. Summed feature 3, C_16:1_ ω7c/C_16:1_ ω6c; Summed feature 5, iso-C_17:1_ω9c, C_18:2_ ω6,9c, ante-C_18:0_; Summed feature 8, C_18:1_ω7c/C_18:1_ω6c; Summed feature 9, iso-C_17:1_ω9c/C_16:0_10-methyl.

Phylogenetic analysis based on 16S rRNA gene sequences placed strains GXAS 306^T^ and GXAS 311 in the family Pleioneaceae. Strains GXAS 306^T^ and GXAS 311 were most closely related to the type strains of the recognized species of the genus *Aliikangiella* and formed a distinct phylogenetic lineage. The phenotypic properties of strains GXAS 306^T^ and GXAS 311 confirmed that they belonged to the genus *Aliikangiella*. However, because of differences in molecular and phenotypic characteristics, strains GXAS 306^T^ and GXAS 311 are considered to represent a novel species of the genus *Aliikangiella*, for which the name *Aliikangiella maris* sp. nov. is proposed. Further experiments will be conducted on the *Aliikangiella maris* sp. nov., marked as strain GXAS 306^T^.

### 3.2 Utilization of nitrogen and phosphorus on strain GXAS 306^T^

#### 3.2.1 Abilities of degrading organic nitrogen

**Figure 2:**
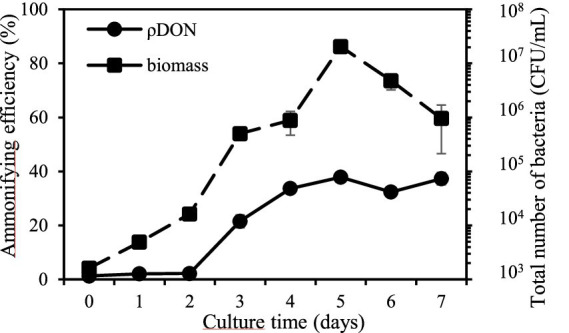
Ability of ammonification in strain GXAS 306^T^.

Genome analysis indicated that strain GXAS 306^T^ had complex metabolic pathways for nitrogen utilization, especially in organic nitrogen metabolism. We thus designed an experiment to test the degradation activity of the strain toward dissolved organic nitrogen (DON), and its utilization of DON in the presence of dissolved inorganic nitrogen (DIN). The results revealed that the strain grew well in DON medium, with maximum biomass (approximately 2 × 10^7^ CFU/mL) and ammonifying efficiency (37.9%) on day 5 culture (Figure 2). This indicated that strain GXAS 306^T^ can break down complex organic nitrogen compounds into simpler forms (e.g., NH_4_^+^-N).

According to the NH^4+^-N content and DON reduction rate analysis, the presence of DIN appeared to increase the bacterial utilization of DON, despite minimal alterations to the biomass compared to the control group without DIN (Supplementary Figure S6A). The NH_4_^+^-N content and DON reduction rate in the control and test groups remained at a relatively low level on day 3 of culture. As culture progressed, a significant increase occurred in its ammonification. The highest content of NH_4_^+^-N in the NO_3_^−^-N and NH_4_^+^-N test groups was 52.7 ± 6.5 and 98.2 ± 5.6 mg/L (Supplementary Figure S6B), which was 34.7 and 150.9% higher than the control group, respectively. The corresponding highest DON reduction rate was 81.0 and 51.4%, which demonstrated a significant enhancement compared to the control group (37.9%) (Supplementary Figure S6C). This addition of NO_3_^−^-N and NH_4_^+^-N highlighted the stimulatory effect of DIN on its ammonification. Similarly, Yang et al. (2007) finds that high concentrations of NH_4_^+^-N stimulate the growth rate of ammonifying bacteria. Interestingly, the delayed increase in NH_4_^+^-N content and DON reduction rate in the NH_4_^+^-N test group revealed a sequential nitrogen utilization strategy employed by strain GXAS 306^T^.

Genomic analyses unveiled a comprehensive suite of genes and gene clusters in strain GXAS 306^T^ that are integral to ammonia assimilation, nitrogen dissimilation, and ammonification (Supplementary Table S4). Nitrogen assimilation is a cornerstone for bacterial growth and maintenance of nitrogen balance, whose rate was bacterial count dependent (Shpigel et al., 2019). Under nitrogen limitation, the activation of NtrC regulatory proteins upregulates genes involved in glutamine synthetase (GS) and glutamate synthase (GOGAT) pathways (North et al., 2023). This regulatory mechanism ensures that the organism optimally utilizes the scarce nitrogen resources available, thereby preserving cellular nitrogen equilibrium. Conversely, nitrogen dissimilation represents another nitrogen metabolism toolkit of strain GXAS 306^T^, serving as a strategic counterbalance to assimilation. In nitrogen-rich environments, the dissimilation pathway assists in mitigating the effects of excessive nitrogen accumulation. Moreover, this pathway contributes to energy generation, which can indirectly bolster the ammonia assimilation processes when nitrogen becomes limiting again. The other nitrogen metabolism pathway of strain GXAS 306T performs ammonification, which bridges organic to inorganic nitrogen and plays a key role in nitrogen cycle. Ammonification is ultimately controlled by microbial functional genes, including related extracellular enzyme genes and intracellular deaminase genes. Among them, partial genes encoding related enzymes are found in ammonifying microbes, such as alkaline metallopeptidases (*apr*, EC:3.4.24), neutral metallopeptidases (*npr*, EC:3.4.24), and serine peptidases (*sub*, EC:3.4.21) (Bach et al., 2000), which were harbored in strain GXAS 306^T^ (Supplementary Table S4). In particular, strain GXAS 306^T^ owned an enzyme (EC:3.5.5.1) related to the degradation of nitriles, which is known for their cytotoxic properties and frequently encountered in plants. In aquatic environments, nitrile degradation is not only a detoxification process for algae and bacteria but also the degraded carboxylic acids and ammonia can provide carbon and nitrogen sources for algae (Egelkamp et al., 2017). Owing to the coexistence of these nitrogen metabolism pathways, strain GXAS 306^T^ survival and proliferation are guaranteed in environments where nitrogen availability oscillates dramatically.

#### 3.2.2 Phosphorus solubilizing characteristics

**Figure 3:**
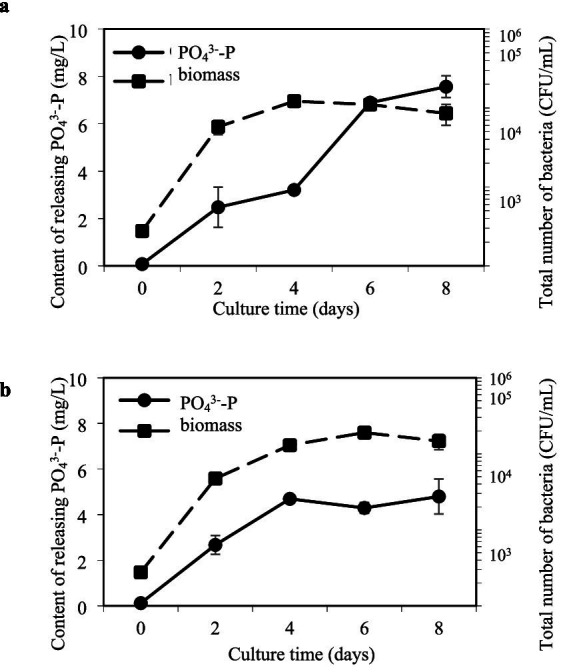
Ability of dissolving insoluble organic phosphate **(A)** and inorganic phosphate **(B)** in strain GXAS 306^T^.

Ammonifying bacteria also have a multitude of beneficial activities (e.g., proteolysis, phosphorus and potassium solubilization, and production of plant hormones) that contribute to plant health and vitality (Przemieniecki et al., 2015; Liu et al., 2014; De Andrade et al., 2023). Among these activities, phosphorus-solubilizing function was found in strain GXAS 306^T^, which was evidenced by its growth in a phosphorus-solubilizing medium and the formation of transparent circles around bacterial colonies (Supplementary Figure S7). The effective phosphate concentrations in organic phosphorus (OP) and inorganic phosphorus (IP) tests increased by 7.6 ± 0.4 mg/L and 4.8 ± 0.6 mg/L on day 8 of culture, respectively (Figure 3). Phosphorus solubilization occurs through a combination of mechanisms, involving both the secretion of phosphatases and the production of organic acids (Prabhu et al., 2019). Alkaline phosphatase is an important organic phosphorus hydrolase in aquatic environments, whose coding genes are divided into three types: *phoA*, *phoD*, and *phoX*. *PhoA* targets phosphate monoesters, whereas *pho*D and *pho*X are active against both phosphate monoesters and diesters (Luo et al., 2009; Rawat et al., 2020). Remarkably, strain GXAS 306^T^ harbored all three *pho* genes (Supplementary Table S4), enabling a versatile response to phosphorus limitation through enhanced expression of these enzymes. In Gram-negative bacteria, this mineral phosphate-solubilization phenotype is also attributed predominantly to the secretion of low molecular weight organic acids, with gluconate being the principal acid described. Glucose dehydrogenase (GDH) catalyzes the oxidation of glucose to gluconic acid, requiring the pyrroloquinoline quinone (PQQ) cofactor for its activity (Sashidar and Podile, 2010). Strain GXAS 306^T^ possessed the genetic toolkit necessary for gluconic acid biosynthesis, featuring genes encoding GDH (*gdh*, EC:1.1.5.9) and PQQ synthase (*pqqC*, EC:1.3.3.11) (Supplementary Table S4). The *pqq*C gene not only facilitates gluconic acid production but also aids in the conversion of iron/aluminum-bound phosphorus to calcium-bound phosphorus, expediting the dissolution of recalcitrant phosphorus in sediments (Ludueña et al., 2017). Hence, the adept navigation of strain GXAS 306^T^ in phosphorus solubilization highlights its pivotal role in the intricate web of nutrient cycling processes.

### 3.3 Interactions of *Phaeocystis globosa* and strain GXAS 306^T^

#### 3.3.1 Utilization of *Phaeocystis globosa* extracellular products

Cultivation of strain GXAS 306^T^ in the filtrate of algal culture unveiled fascinating insights into its capacity to assimilate and metabolize complex organic substrates in the phycosphere. The analysis of the filtrate indicated that dissolved organic carbon (DOC) was consumed. The content of DOC was 26.8 ± 1.1 mg/L on day 7 culture, which was 22.8% lower than that of initial content (36.5 ± 0.8 mg/L). The data of annotated genes by the CAZy database showed that the strain has a suite of enzymes including glycoside hydrolases (15) and carbohydrate esterases (27), which actively assimilate dissolved organic carbon for its metabolic processes. Such a capacity for utilizing DOC reflects not only the metabolic flexibility of strain GXAS 306^T^ but also its competitive edge in resource-limited marine environments.

#### 3.3.2 Influence of strain GXAS 306^T^ on the growth of *Phaeocystis globosa*

Recent findings from our team reveal intriguing differences in nutrient preference, substance production, and energy allocation strategies exhibited by two *P. globosa* strains Pg293 and PgV01, especially in nutritional limitation (Li J. et al., 2023; Fu et al., 2024). Compared to Pg293, PgV01 showcases a heightened sensitivity to nitrogen and phosphorus enrichment on early growth, with a notably faster growth rate. Upon exposure to nutrient stress, both strains exhibit increased production of extracellular polysaccharides and total exopolymeric substances in the initial growth phase. Given the distinctive physiological profiles of Pg293 and PgV01, we set out to explore how the introduction of strain GXAS 306^T^ influences their growth kinetics and physiological states.

**Figure 4:**
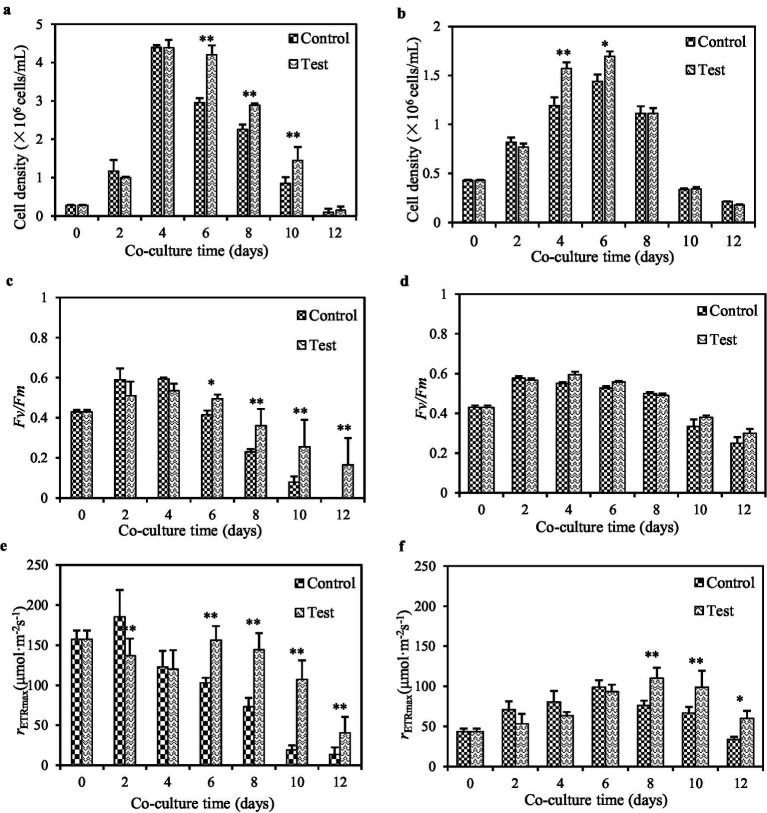
Influences of strain GXAS 306^T^ in algal growth parameters of Pg293 **(A,C,E)** and PgV01 **(B,D,F)**. Control, the pure culture of PgV01 and Pg293; Test, co-culture of strain GXAS 306^T^ and *P. globosa*. Error bars indicate standard deviations for the three replicates. * and ** indicate the significant differences between the control and the tests at the levels of *p* < 0.05 and *p* < 0.01, respectively.

Under ample nutrient conditions, both Pg293 and PgV01 exhibited a 12-day growth cycle (Figures 4A,B). The peak cell density reached 4.4 × 10^6^ cells/mL for Pg293 on day 4 and 1.4 × 10^6^ cells/mL for PgV01 on day 6. Beyond mere cell counts, the health status and stress responsiveness of these algae were further elucidated by monitoring key photosynthetic parameters *F*_v_*/F*_m_ and *r*_ETRmax_. Both Pg293 and PgV01 exhibited comparable trends in these parameters (Figures 4C–F). In particular, the *F*_v_*/F*_m_ of Pg293 on day 8 plummeted beneath the crucial point (0.3) (Figure 4C), which signifies a downturn in algal health and vitality (Millar et al., 2024). Conversely, PgV01 exhibited superior tenacity, maintaining its *F*_v_*/F*_m_ above the crucial point until day 12 (Figure 4D). Meanwhile, the *r*_ETRmax_ for both strains showed crucial points at days 2 and 6, respectively, with crucial points of 185.7 and 99.0 μmol photons m^−2^ s^−1^ (Figures 4E,F). These points serve as indicators of nutritional limitation, signaling the onset of stress-induced modifications in the algal metabolic strategies (Sun et al., 2019). Upon introduction of strain GXAS 306^T^, a notable shift in growth dynamics and photosynthetic parameters of both Pg293 and PgV01 was observed. For Pg293, the intervention led to a pronounced increase in cell counts starting from the late growth period (*p* < 0.01), with an average growth rate of 19.6% (Figure 4A). Concurrently, there was a significant enhancement in *F*_v_*/F*_m_, delaying its descent below the crucial point until day 8 (Figure 4C). The crucial point of *r*_ETRmax_ was similarly postponed to day 6, settling at 155.9 photons m^−2^ s^−1^ (Figure 4E). For PgV01, the effect of cell counts was a significant increase in logarithmic growth period (*p* < 0.01, *p* < 0.05), with an average growth rate of 10.1% (Figure 4B). There were no statistically significant variations in *F*_v_*/F*_m_ throughout the growth cycle (*p* = 0.37) (Figure 4D). The *r*_ETRmax_ witnessed a substantial uplift in the later phase of growth (*p* < 0.01), and its crucial point (109.9 μmol photons m^−2^ s^−1^) was deferred to day 8 (Figure 4F). Although the impact on both Pg293 and PgV01 was different, the presence of strain GXAS 306^T^ promoted their growth and improved photosynthetic efficiency. The growth and reproductive processes of *P. globosa* involve substantial consumption of inorganic nitrogen and inorganic phosphorus, with copious production of exopolymers rich in polysaccharides, proteins, nucleic acids, and lipids (Solomon et al., 2003). As reported in literature, the provision of essential nutrients near the stationary phase of the culture can produce positive outcomes in cell counts and photosynthetic indices (Rodríguez-Román and Iglesias-Prieto, 2005; Parkhill et al., 2001; Browning et al., 2014). Decomposition of algal extracellular organic matter (e.g., organic nitrogen and phosphorus) by strain GXAS 306^T^ may provide inorganic nutrients (e.g., nitrogen and phosphorus) for the algal growth. In particular, bacterial uptake and utilization of organic phosphorus show a marked increase in phosphorus-limited settings. Only half of the inorganic phosphorus derived from decomposition is typically taken up by bacteria, leaving residual inorganic phosphorus in the aqueous environment (Bjorkman and Karl, 1994).

Beyond improving microenvironment around the alga by degradation of algal extracellular organic matter, phycosphere bacteria can also impact algal growth by releasing substances, including vitamins, trace elements, and growth-promoting factors (Wang et al., 2016). The data of annotated genes (Supplementary Table S5) from the KEGG database indicated that strain GXAS 306^T^ could potentially secrete many plant growth hormones, such as indole-3-acetic acid, biotin, and polyamine, and various fat-soluble and water-soluble vitamins, such as vitamins B_1_, B_2_, B_6_, B_9,_ and B_12_. Among them, vitamins B_12_ and B_1_ are crucial for amino acid and carbohydrate metabolism in algal cells (Bertrand and Allen, 2012). Bacteria in co-culture with *P. globosa* relieve nutrient stress when B vitamins are withheld (Brisbin et al., 2022). In particular, strain GXAS 306^T^ possessed catalase activity capable of degrading hydrogen peroxide (H_2_O_2_), which is a potent oxidant produced abundantly during algal photosynthesis and photorespiration (Petrov and Van Breusegem, 2012). By neutralizing H_2_O_2_, heterotrophic bacteria protect algae against oxidative stress (Morris et al., 2011), further supporting healthy algal growth under stress. Collectively, our results together with the data from literature suggested that strain GXAS 306^T^ might also provide beneficial compounds to stimulate algal growth and potentiate photosynthetic machinery efficiency.

#### 3.3.3 Influence of strain GXAS 306^T^ on *Phaeocystis globosa* growth under P deficiency

Considering the pivotal role of dissolved inorganic phosphorus (DIP) in the lifecycle of *P. globosa* bloom, and given its chronic scarcity in Beibu Gulf, exploring the influence of strain GXAS 306^T^ on the growth dynamics of *P. globosa* under phosphorus deficiency assumes considerable significance. Under dissolved PO_4_^3−^-P deficiency, the growth period of *P. globosa* was drastically shortened to 6 days, with cell counts plummeting by nearly 65.1–77.3% compared to that in nutrient-abundant conditions (Supplementary Figures S8A,B). This nutrient deficiency triggered a cascading failure in the algal physiological functions, culminating in a sharp nosedive in *F*_v_*/F*_m_, wherein that of PgV01 plummet to the detection limit on day 5 (Supplementary Figures S8C,D). Similarly, *r*_ETRmax_ of Pg293 and PgV01 followed a significant downward trend, reaching their crucial points on days 1 and 3, with values of 178.6 and 88.4 μmol photons m^−2^ s^−1^, respectively (Supplementary Figures S8E,F). These observations resonate with established knowledge regarding emergency phosphorus supply systems and regulatory mechanisms of *P. globosa* under phosphorus stress (Lim et al., 1996). As phosphorus stress persists, the depletion of internal reserves leads to a drastic drop in cell counts and algal photosynthetic performance. Upon the introduction of strain GXAS 306^T^, the growth trajectory of *P. globosa* under phosphorus deficiency was markedly altered. They resulted in a significant increase in cell density for both Pg293 and PgV01, with a mean density of 4.8 × 10^5^ and 5.3 × 10^5^ cells/mL, respectively, representing enhancements of 55.2 and 83.5% (Supplementary Figures S8A,B). The impact of strain GXAS 306^T^ on algae under phosphorus deficiency was significantly greater than that under nutrient-abundant conditions (with increases of 19.6 and 10.1%, respectively, in the latter case). Beyond mere quantitative improvements, the intervention of strain GXAS 306^T^ significantly ameliorated the photosynthetic efficiency of *P. globosa*, particularly in the late growth period (*p* < 0.01). The time, when *Fv/Fm* dropped to the critical point, was postponed to day 4 for Pg293 and day 6 for PgV01 (Supplementary Figures S8C,D). *r*_ETRmax_ of both strains was maintained at increased levels in the late growth period (*p* < 0.01) (Supplementary Figures S8E,F).

Under phosphorus deficiency, the strain GXAS 306^T^ showcased remarkable capabilities in enhancing the growth of *P. globosa*, outperforming growth-promoting capabilities in the standard algal culture mediums significantly. Given the multifactorial nature of algal growth promotion, it might be speculated that the phosphorus solubilization function of strain GXAS 306^T^ took center stage in the test. This hypothesis aligned with existing literature that links phosphorus-solubilizing bacteria to *P. globosa* bloom, emphasizing their crucial role in sculpting phosphorus-solubilizing microbial communities (Tu et al., 2024; Das et al., 2007; Qian et al., 2010). Despite this awareness, empirical validation of fostering algal growth by phosphorus-solubilizing bacteria in P-deficient environments remains limited. Strain GXAS 306^T^ joined a list of bacteria known to promote algal growth under phosphorus deprivation, alongside genera such as *Mucilaginibacter* (Xiao et al., 2024), *Citricoccus* (Zhang et al., 2016), *Devosia* (Deng et al., 2024), *Mycolicibacterium* (Xiao et al., 2022a), *Nocardioides* (Xiao et al., 2022b), *Sphingomonas* (Wang et al., 2024), and others from the phyla Bacteroidetes and Proteobacteria (Xiao L. et al., 2022). These results suggested that strain GXAS 306^T^ stimulates the growth of *P. globosa* under phosphorus deficiency. This effect is dynamic and complex and may be related to the phosphorus-solubilizing activity of strain GXAS 306^T^.

#### 3.3.4 Influences of strain GXAS 306^T^ on *Phaeocystis globosa* growth under N deficiency

Given the high dependence of *P. globosa* on nitrogen during growth, we further explore the co-culture of strain GXAS 306^T^ with *P. globosa* and their interactions under nitrogen deficiency. When subjected to available nitrogen (NO_3_^−^-N) deficiency, the growth cycle of *P. globosa* was shortened to 6–8 days, and cell counts were reduced by 67.1–86.0% compared to that in nutrient-abundant conditions (Supplementary Figures S9A,B). This nitrogen deficiency further affected the physiological wellbeing of *P. globosa*, reflecting precipitous declines in *F*_v_*/F*_m_ and *r*_ETRmax_ (Supplementary Figures S9C–F). Despite the challenges imposed by nitrogen deficiency, the introduction of strain GXAS 306^T^ proved advantageous. The presence of strain GXAS 306^T^ facilitated a significant boost in cell counts, recording increments of 19.8% for Pg293 and 68.9% for PgV01 (Supplementary Figures S9A,B). Moreover, strain GXAS 306^T^ played a protective role in stabilizing *F*_v_*/F*_m_ and *r*_ETRmax_ of both strains across the entire experimental duration, with statistical significance (*p* < 0.01) during the mid-to-late growth period (Supplementary Figures S9C–F).

Nitrogen deficiency severely limits the growth of *P. globosa*. However, the presence of strain GXAS 306^T^ introduced a mitigative dimension, significantly impacting *P. globosa* during the late growth period. It might be speculated that the ammonifying function of strain GXAS 306^T^ played an important role in the algal growth-promoting effect. Despite an absence of concrete evidence indicating direct interactions between surface-dwelling ammonifying bacteria and *P. globosa*, the current comprehension of ammonifying bacterial activities establishes a theoretical framework for potential symbioses. In aquatic environments, the majority of plants and microorganisms lack the capability to directly assimilate nitrogenous organic matter in its complex form. The nitrogenous organic matter requires to be converted into simpler, bioavailable forms through microbial degradation, which is a critical process known as mineralization (Zhao et al., 2015). Ammonifying bacteria, as one of the pieces of evidence for the active ammonification of nitrogen-containing organic compounds (Podlesnaya et al., 2021), whose distribution and ammonifying efficiency are positively correlated with the levels of physicochemical parameters such as dissolved organic nitrogen (DON), inorganic salts (NH₄^+^-N), and dissolved oxygen (DO) (Davies et al., 1995; Juhna et al., 2007; Yang et al., 2007). Based on the abundant DON, DO and NH₄^+^-N, these aquatic environments fostered a conducive setting for ammonifying bacteria to proliferate and recycle nutrients. Correspondingly, an equivalent optimal ecological niche emerges during *P. globosa* blooms. The metagenomic analysis revealed that heterotrophic bacteria are adept at utilizing dense nutrient sources (e.g., organic phosphorus), and breaking down intricate proteins and polysaccharides emitted by *P. globosa*, securing notable competitive advantages (Gibson et al., 2022; Xu S. et al., 2022; Shi et al., 2023). The role of ammonifying bacteria in the growth and dissipation of *P. globosa* bloom deserves further investigation in the future. Intriguingly, nitrogen selectivity of *P. globosa* complicates matters, showing an initial preference for nitrate and urea, with an amplified need for ammonium upon encountering nitrogen scarcity (Wang et al., 2011). Based on our results, strain GXAS 306^T^ has a positive impact on the growth of *P. globosa* under nitrogen deficiency, and the effect may be related to bacterial ammonifying function depending on environmental changes and the factors intrinsic to algae.

### 3.4 Description of *Aliikangiella maris* sp. nov.

*Aliikangiella maris* (ma’ris. L. gen. Neut. n. *maris*, of the sea, isolated from the seawater).

Cells are Gram-negative, non-spore-forming, aerobic, short rods, motile with single polar flagellum (0.2–0.8 × 1.2–2.8 μm). Colonies on MA plates incubated at 30°C for 3 days are pale yellow, circular with smooth surfaces. Growth occurs at 15–37°C (optimum, 28–30°C), pH 5.5–10.5 (optimum, pH 6–7), and with NaCl concentrations of 0–4% (w/v) (optimum, 2–3%). Cells are positive for catalase, casein, Tween-20 and Tween-80 hydrolyzation, and peptonization of milk, but negative for the production of H_2_S, oxidase activity, and hydrolysis of cellulose and starch. The predominant quinone is ubiquinone Q-8. The major fatty acids are iso-C_15:0_, C_14:0_ DMA, and summed feature 9. The polar lipids are found to contain diphosphatidylglycerol (DPG), phosphatidylglycerol (PG), phosphatidylethanolamine (PE), unknown aminolipids (AL), unknown glycolipids (GL), unidentified amino phospholipid (APL), and unknown lipid (L). The G + C content of the draft genome of the type strain is 38.5 mol%.

The type strain, GXAS 306^T^ (=MCCC 1K08359^T^ = KCTC 92831^T^), was isolated from a surface seawater sample in Qinzhou Bay, Guangxi Zhuang Autonomous Region, China. The GenBank accession number of the type strain 16S rRNA gene sequence is PP905594, and that of the draft genome is JBFDAH000000000.

## 4 Conclusion

A novel species (type strain GXAS 306^T^) of the genus *Aliikangiella* was isolated from the phycosphere of *P. globosa* bloom, and we established *Aliikangiella maris* sp. nov. based on the polyphasic taxonomic study. Genomic analysis indicated that strain GXAS 306^T^ contained multiple functions related to interactions with algae and bacteria, among which its ammonifying and phosphorus-solubilizing function was further validated. Strain GXAS 306^T^ had a positive effect on the growth of *P. globosa*, which was also reflected under nitrogen and phosphorus deficiency. Our study provided novel information on the interaction between phycosphere bacteria and *P. globosa*. This investigation serves as a stepping stone toward a deeper comprehension of the interplay between nutrients, *P. globosa*, and bacteria in marine ecosystems.

## Data Availability

The datasets presented in this study can be found in online repositories. The names of the repository/repositories and accession number(s) can be found in the article/Supplementary material.

## Footnotes

^1^ https://lpsn.dsmz.de/genus/aliikangiella

^2^ http://soap.genomics.org.cn/soapdenovo.html

^3^ https://automlst.ziemertlab.com/

^4^ https://cloud.majorbio.com/page/tools/

^5^ http://ggdc.dsmz.de/
