# Supplementary material for: Ammonifying and phosphorus-solubilizing function of Aliikangiella maris sp. nov. isolated from Phaeocystis globosa bloom and algal–bacterial interactions
Source: Front Microbiol. 2025 Feb 10;16:1516993. doi: 10.3389/fmicb.2025.1516993 (PMC11849500; doi:10.3389/fmicb.2025.1516993)
Supplement: Supplementary file 1 [file Data_Sheet_1.PDF]

Supplementary materials

Figure S1. Transmission electron micrograph of cells of strains GXAS 306^T (a) and GXAS 311(b) grown on MA for 3 days at 30 °C. Bar, 500 nm.

a

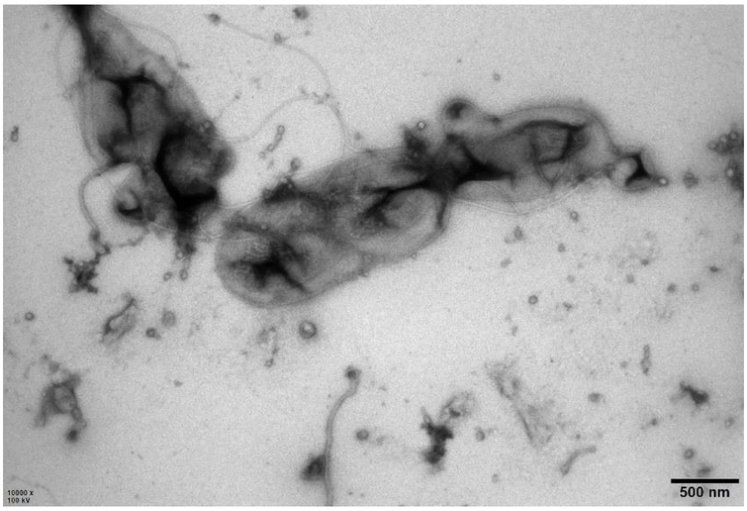

b

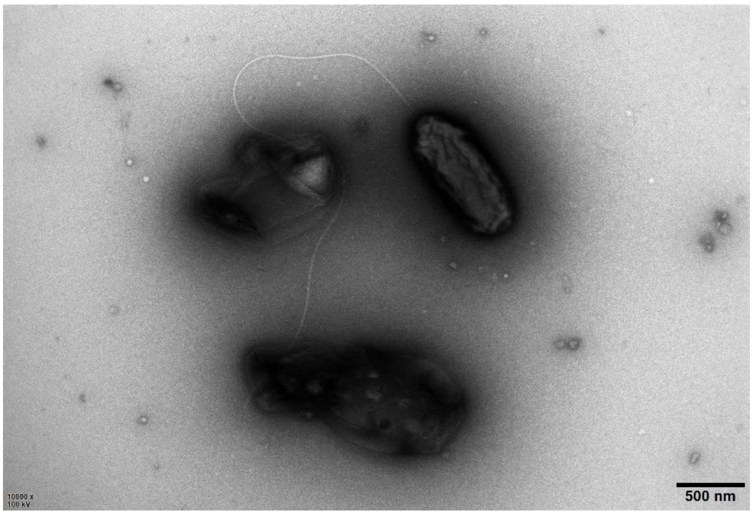

Figure S2. Two-dimensionalthin layer chromatographs showing the polar lipids of strains GXAS 306^T (a-d). (a) molybdatophosphoric acid reagent, (b) molybdenum blue reagent, (c) ninhydrin reagent, (d) anisaldehyde reagent. DPG, diphosphatidylglycerol; PE, phosphatidylethanolamine; PG, phosphatidylglycerol; AL, unknown aminolipid; APL, unidentified amino phospholipid(s); GL, unknown glycolipid(s); L, unidentified polar lipid.

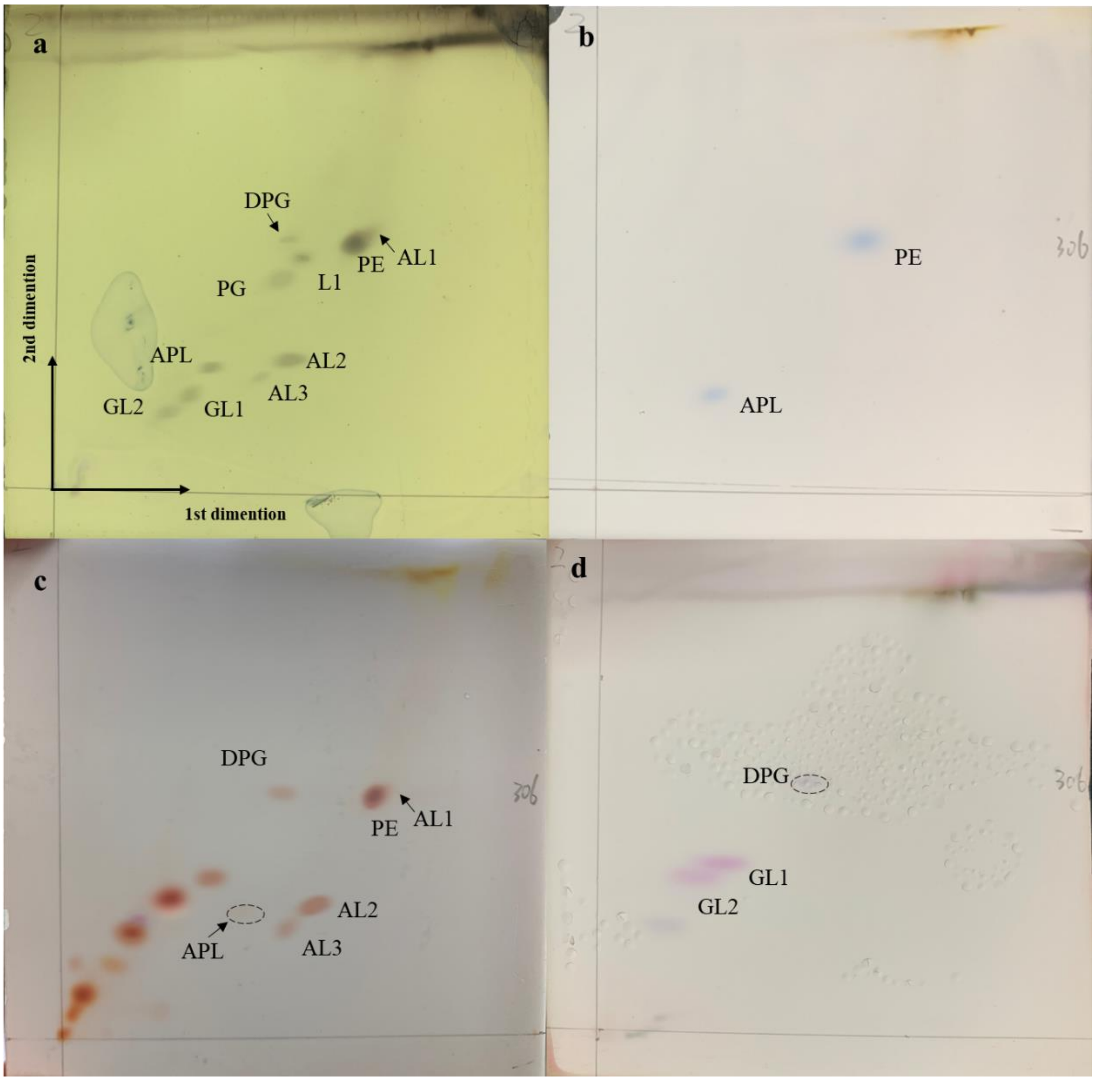

Figure S3. Neighbour-joining phylogenetic tree based on 16S rRNA gene sequences showing the relationships between strain GXAS 306^T and closely related taxa. Scale bar indicates 0.02 substitutions per nucleotide position. *Escherichia coli* ATCC 11775^T(X80725) was used as outgroup. GenBank accession numbers for each 16S rRNA gene sequence are listed in parentheses.

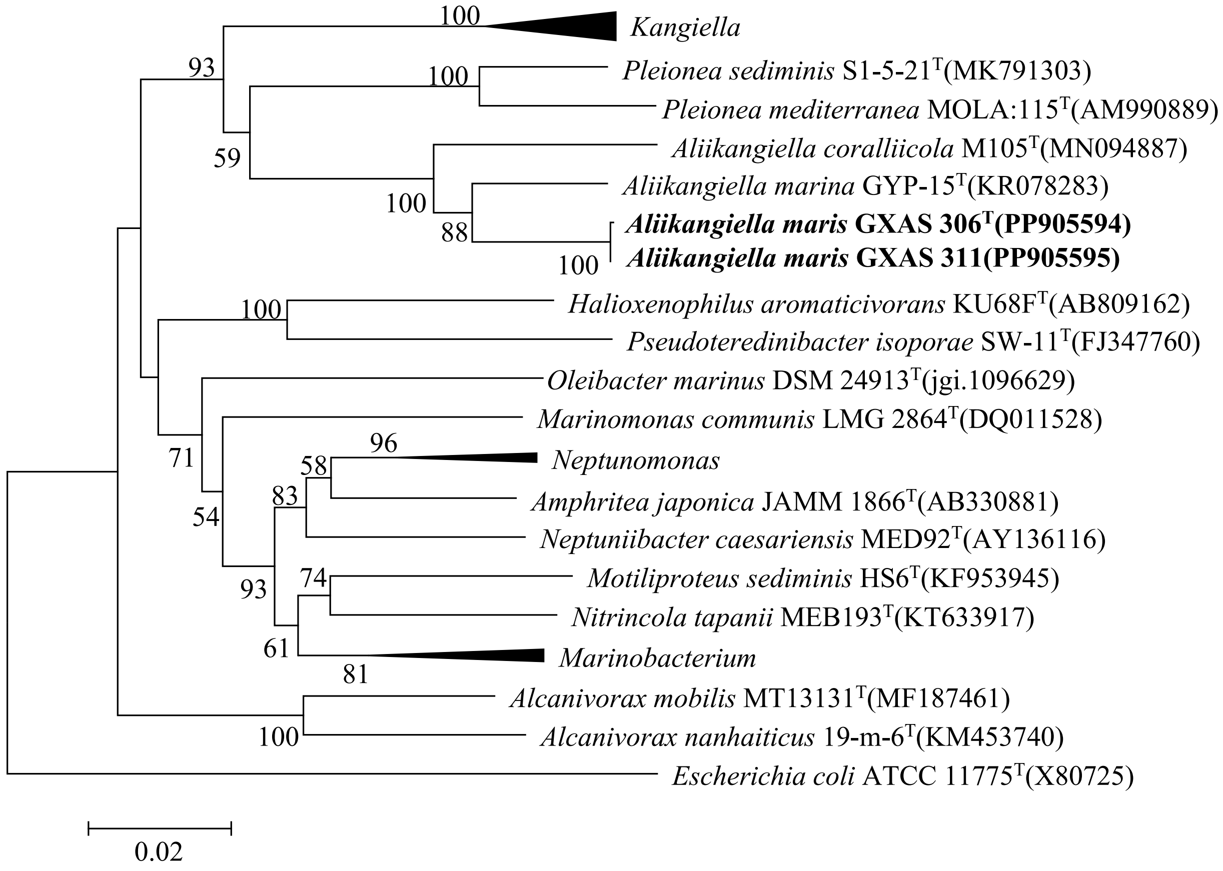

Figure S4. Maximum-parsimony phylogenetic tree based on 16S rRNA gene sequences showing the relationships between strain GXAS 306^T and closely related taxa. GenBank accession numbers for each 16S rRNA gene sequence are listed in parentheses.

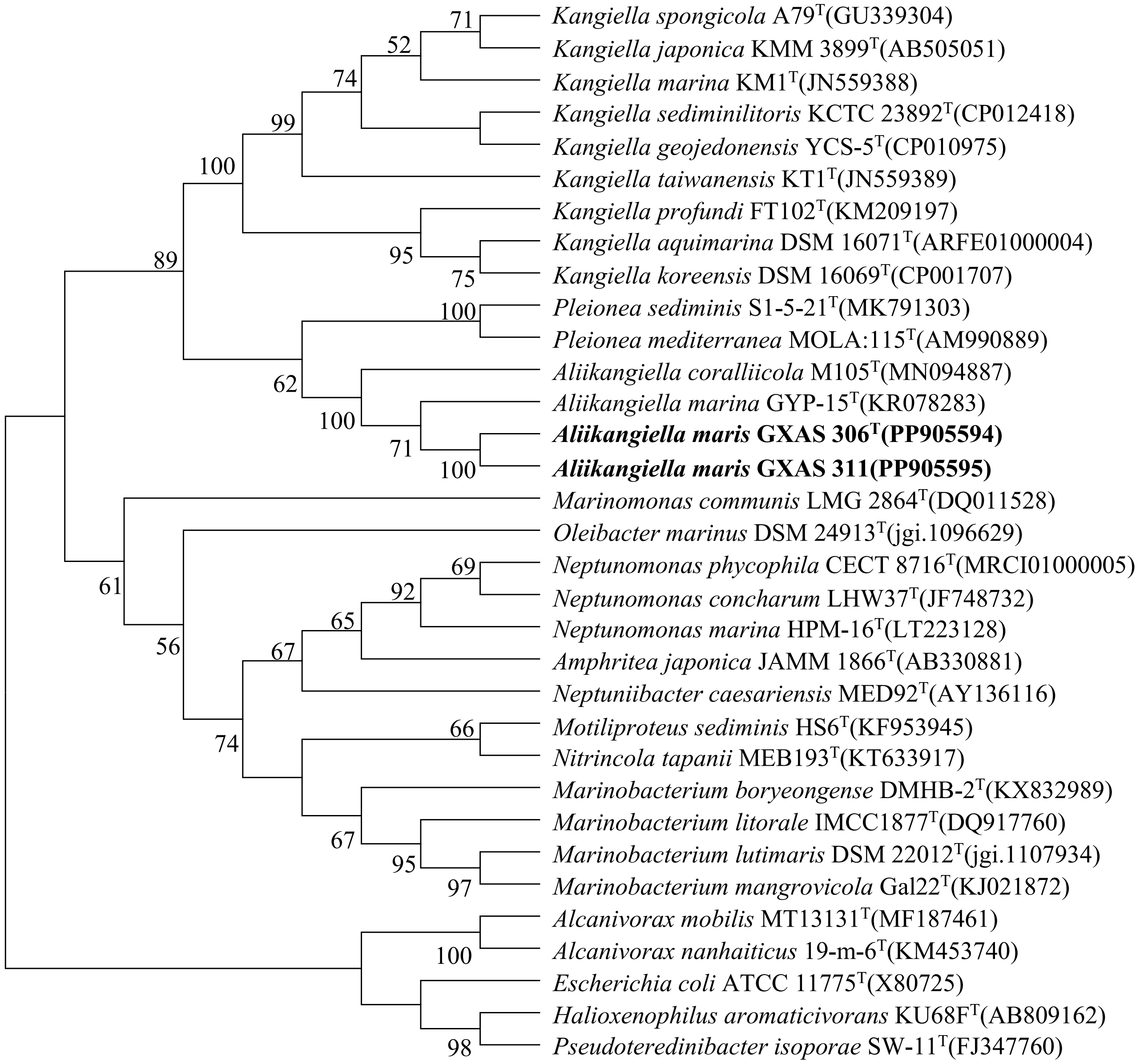

Figure S5. Maximum-likelihood phylogenetic tree based on 83 housekeeping genes indicating the phylogenetic position of strain among strains GXAS 306^T and GXAS 311 compared with the related species. Scale bar indicates 0.05 substitutions per nucleotide position. *Bacillus altitudinis* 41KF2b^T (OVSL00000000) was used as outgroup. GenBank accession numbers for each sequence are listed in parentheses.

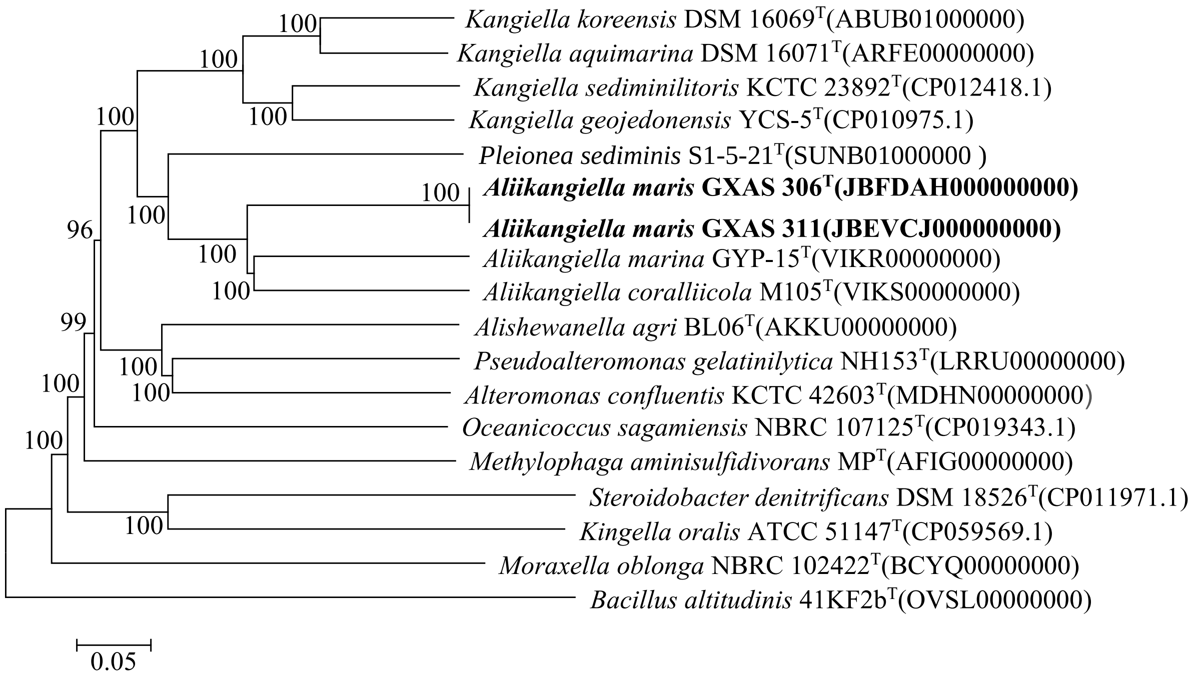

Figure S6. The influence of nitrate and ammonium salts on ammonification of strain GXAS 306^T. "Control", MA medium (without NH_4NO_3). "Test($+NO_3^-$)" and "Test($+NH_4^+$)" indicate the MA medium was supplemented with $NaNO_3$ and $(NH_4)_2SO_4$, respectively.

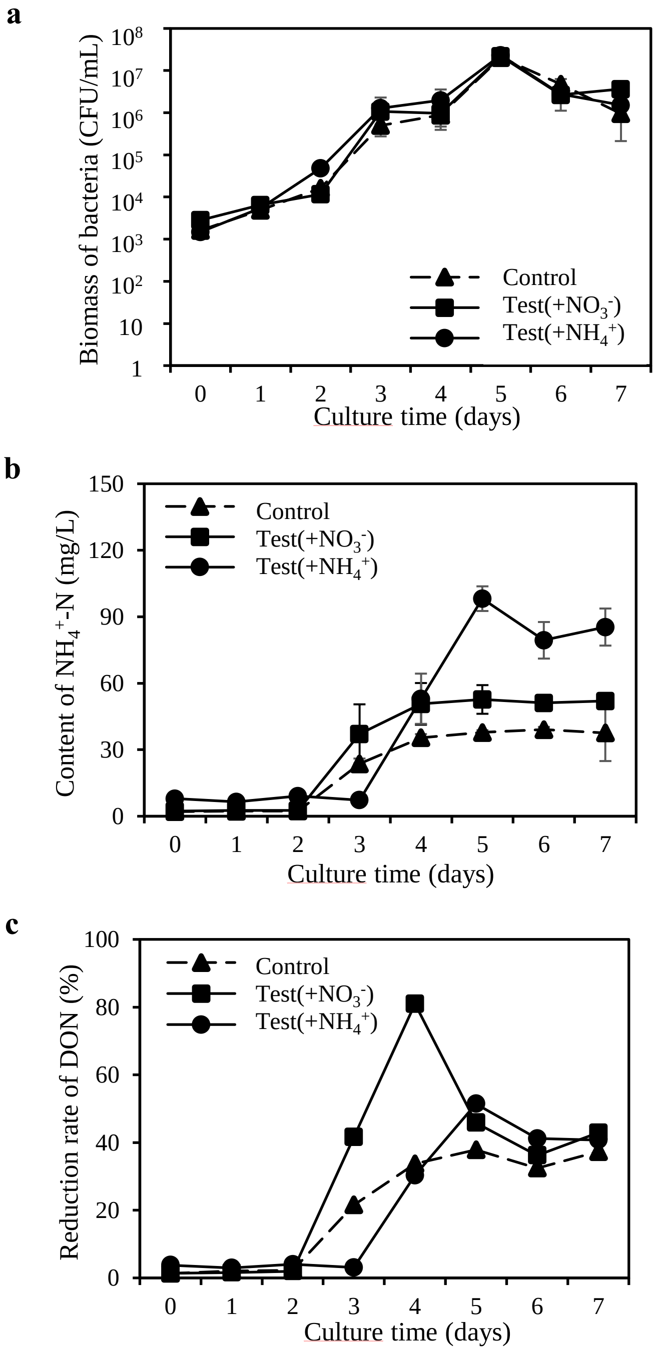

Figure S7. Colony morphology of strain GXAS 306^T grown on phosphate-solubilizing medium for 7 days at 28 °C. (a) organic phosphorus-solubilizing and (b) inorganic phosphorus-solubilizing.

a b

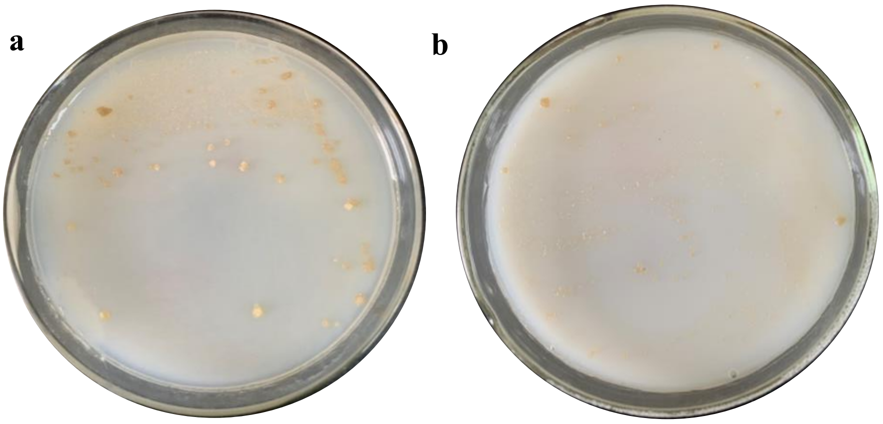

Figure S8. The influences of strain GXAS 306[T] on algal growth parameters of Pg293 (a, c, e) and PgV01 (b, d, f) under P deficiency. The pure culture of PgV01 and Pg293 served as the control. Error bars indicate standard deviations for the three replicates. Control, the pure culture of PgV01 and Pg293; Test, co-culture of strain GXAS 306[T] and *P. globosa*. * and ** indicate the significant differences between the control and the tests at the levels of $P<0.05$ and $P<0.01$, respectively.

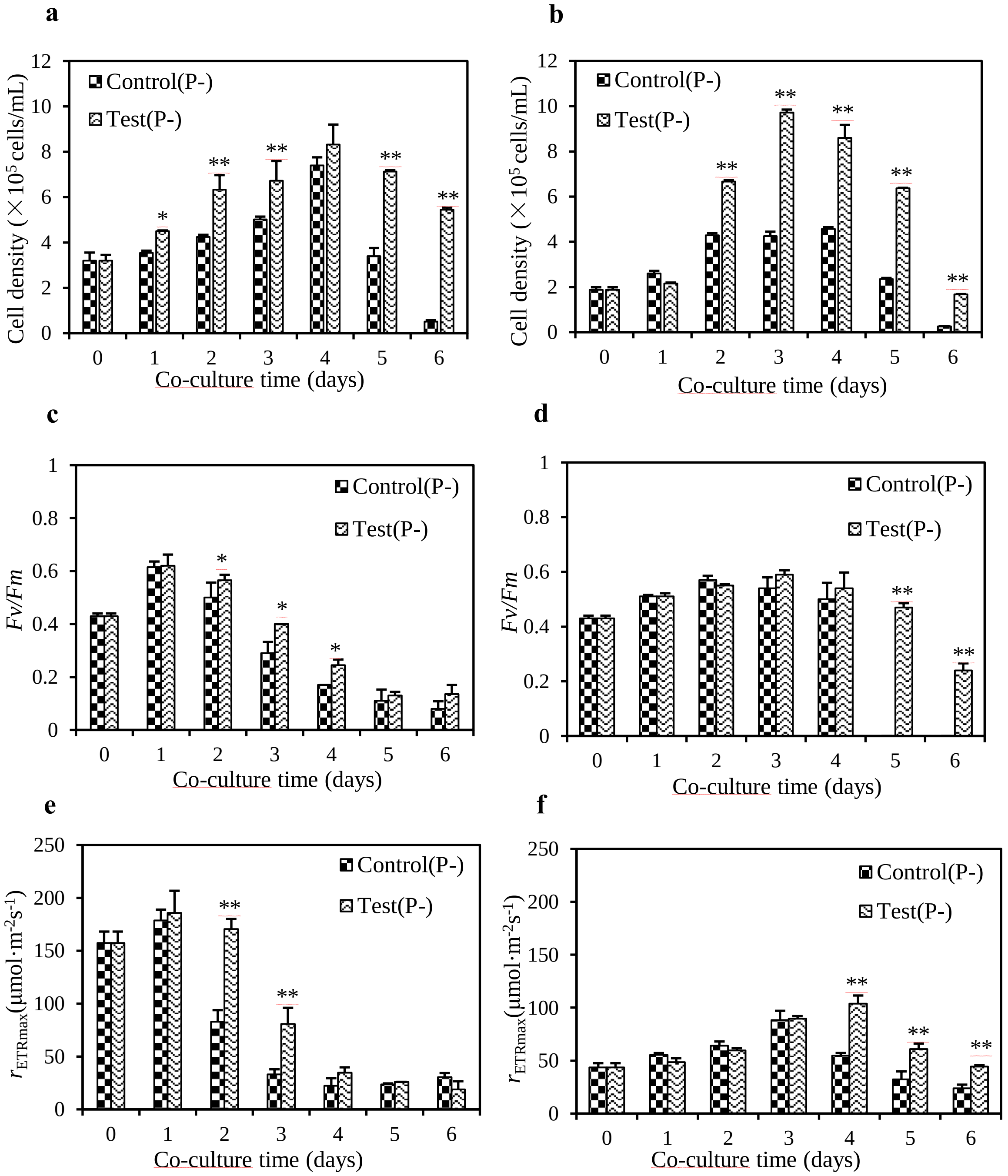

Figure S9. The influences of strain GXAS 306^T on algal growth parameters of Pg293 (a, c, e) and PgV01 (b, d, f) under N deficiency. The pure culture of PgV01 and Pg293 served as the control. Error bars indicate standard deviations for the three replicates. Control, the pure culture of PgV01 and Pg293; Test, co-culture of strain GXAS 306^T and *P. globosa*. * and ** indicate the significant differences between the control and the tests at the levels of P<0.05 and P<0.01, respectively.

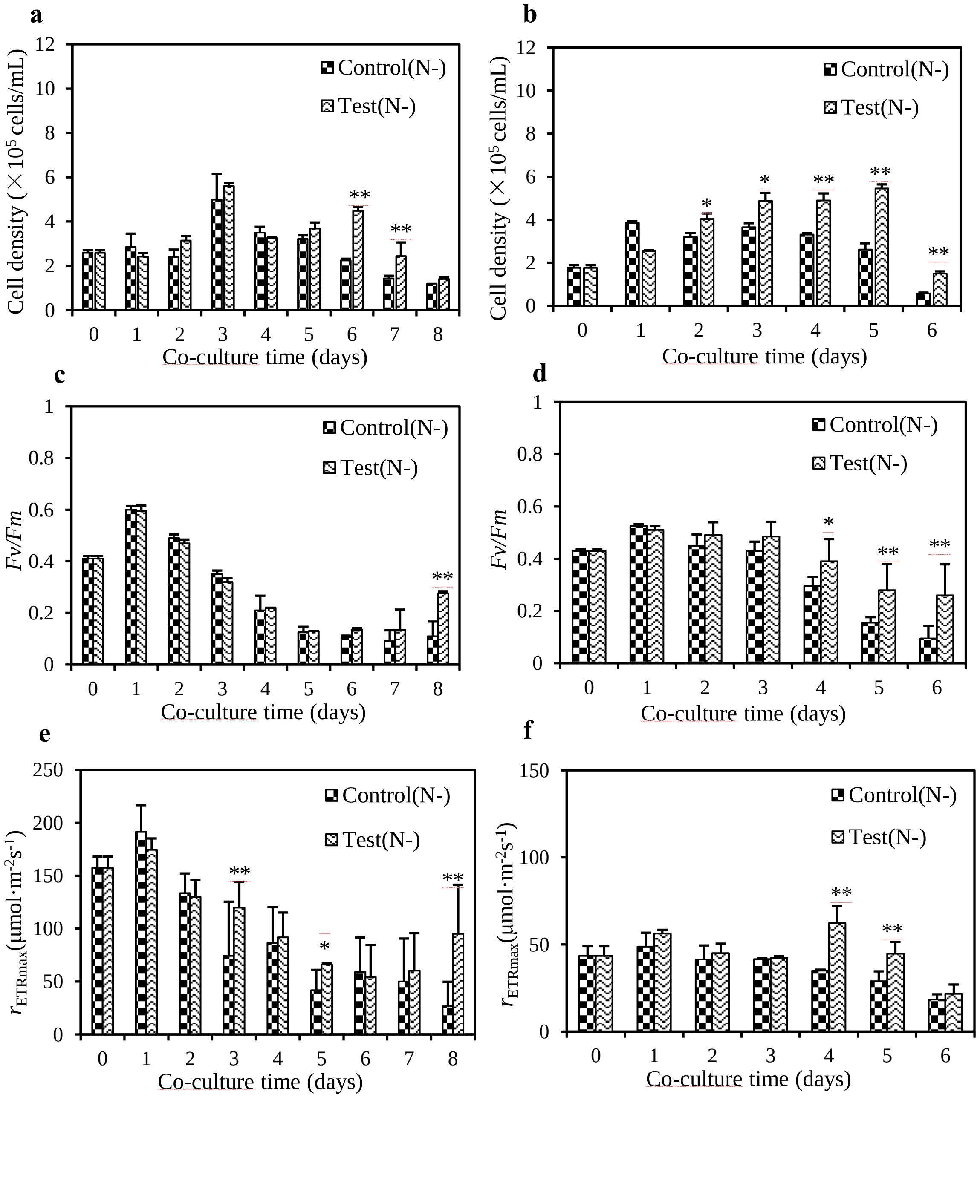

Table S1. General genome features of strain GXAS 306^T and the related species. Strains: 1, GXAS 306^T; 2, GXAS 311; 3, *Aliikangiella marina* GYP-15^T; 4, *Aliikangiella coralliicola* M105^T; 5, *Pleionea sediminis* S1-5-21^T.

**Features**	**1**	**2**	**3**	**4**	**5**
Assembly accession	GCA_040513415.1	GCA_040513415.1	GCA_007004765.1	GCA_007004725.1	GCA_007570825.1
Genome length (bp)	5,379,662	5,049,449	5,336,331	7,044,683	5,194,783
Assembled contigs	118	196	8	22	26
Contig N50 (bp)	186,552	132,530	1,289,802	500,880	917,035
G+C content (mol%)	38.5	38.5	41.9	41.5	40.1
Total genes	4,483	4,264	4,536	5,373	4,470
Protein coding genes	4,392	4,187	4,438	5,287	4,411
rRNA (5S, 16S, 23S)	1, 1, 1	1, 1, 1	3, 3, 3	3, 1, 1	1, 1, 1
tRNA	48	43	56	40	44
ncRNA	4	4	5	5	4
Genome coverage	227.6x	286.8x	217.0x	197.0x	100.0x

Table S2. 16S rRNA gene sequence similarities, average nucleotide identities (ANI), average amino acid identities (AAI) and digital DNA-DNA hybridization (dDDH) values (%) between strain GXAS 306^T and the related species.

**Strain**	**16S similarity (%)**	**ANI (%)**	**AAI (%)**	**dDDH (%)**
Strain GXAS 311	99.7	99.9	100	99.8
Aliikangiella marina GYP-15^T	95.8	69.6	67.0	18.8
Aliikangiella coralliicola M105^T	94.2	69.9	67.0	19.0
Pleionea sediminis S1-5-21^T	90.1	67.5	57.4	17.9
Pleionea mediterranea MOLA:115^T	89.0	67.5	57.3	18.7
Kangiella profundi FT102^T	90.1	67.4	56.6	18.9
Kangiella aquimarina DSM 16071^T	89.8	67.2	56.7	18.0
Kangiella koreensis DSM 16069^T	89.5	67.2	56.4	24.1

Table S3. Unique bacterial gene functions in Strain GXAS 306^T and GXAS 311 among three analyzed strains by RAST. *Aliikangiella marina* GYP-15^T, *Aliikangiella coralliicola* M105^T and *Pleionea sediminis* S1-5-21^T were analyzed.

Category	Subsystem	Role
Amino Acids and Derivatives	Methionine Degradation	2-Oxobutyrate oxidase, putative
	Methionine Biosynthesis	O-acetylhomoserine sulfhydrylase (EC 2.5.1.49)
		O-succinylhomoserine sulfhydrylase (EC 2.5.1.48)
	Proline, 4-hydroxyproline uptake and utilization	Similar to eukaryotic Peptidyl prolyl 4-hydroxylase, alpha subunit (EC 1.14.11.2)
Carbohydrates	Acetyl-CoA fermentation to Butyrate	3-hydroxybutyryl-CoA dehydrogenase (EC 1.1.1.157)
	Pyruvate metabolism II: acetyl-CoA, acetogenesis from pyruvate	NAD-independent protein deacetylase AcuC
	Pyruvate metabolism II: acetyl-CoA, acetogenesis from pyruvate	Phosphate acetyltransferase (EC 2.3.1.8)
Clustering-based subsystems	CBSS-393124.3.peg.2657	ADA regulatory protein
	Bacterial cell division cluster	Cell division topological specificity factor MinE
	CBSS-349161.4.peg.2417	DNA primase (EC 2.7.7.-)
	CBSS-354.1.peg.876	FIG002903: a protein of unknown function perhaps involved in purine metabolism
	CBSS-354.1.peg.2917	FIG003879: Predicted amidohydrolase
	Bacterial cell division cluster	Septum site-determining protein MinC, MinD

Cofactors, Vitamins, Prosthetic Groups, Pigments	Folate Biosynthesis	PqqC-like protein
DNA Metabolism	CRISPRs	CRISPR-associated helicase Cas1/3, Csy1/2/3 family
	DNA repair, bacterial	Exonuclease SbcC
Dormancy and Sporulation	Persister Cells	HipB protein
Fatty Acids, Lipids, and Isoprenoids	Fatty Acid Biosynthesis FASII	Enoyl-[acyl-carrier-protein] reductase [NADH] (EC 1.3.1.9)
Membrane Transport	NhaA, NhaD and Sodium-dependent phosphate transporters	Na+/H+ antiporter NhaD type
Miscellaneous	Phosphoglycerate mutase protein family	Putative periplasmic protein YibQ, distant homology with nucleoside diphosphatase and polysaccharide deacetylase
Protein Metabolism	Protein degradation	Aminopeptidase YpdF (MP-, MA-, MS-, AP-, NP- specific)
Regulons	ar-431-EC Molybdopterin-guanine dinucleotide biosynthesis	Molybdopterin-guanine dinucleotide biosynthesis protein MobA
Stress Response	Synthesis of osmoregulated periplasmic glucans	Glucans biosynthesis protein C (EC 2.1.-.-)
	Glutaredoxins	Glutaredoxin 3
Sulfur Metabolism	Thioredoxin-disulfide reductase	Thiol peroxidase, Tpx-type (EC 1.11.1.15)

Table S4. The possibly partial important genes or gene clusters of strain GXAS 306^T involved in nutrients utilization related to algal-bacterial interaction.

Nutrients utilization	Gene ID	Location	KEGG Name	KO ID	KO Description	Pathway ID
**Nitrate metabolism**						
**Nitrogen dissimilation**	gene1555	Scaffold6	narX	K07673	two-component system, NarL family, nitrate/nitrite sensor histidine kinase NarX [EC:2.7.13.3]	ko02020
	gene1556	Scaffold6	narL	K07684	two-component system, NarL family, nitrate/nitrite response regulator NarL	ko02020
	gene1552	Scaffold6	narG	K00370	nitrate reductase / nitrite oxidoreductase, alpha subunit [EC:1.7.5.1 1.7.99.-]	ko00910; ko02020; ko01100; ko01120
	gene1551	Scaffold6	narH	K00371	nitrate reductase / nitrite oxidoreductase, beta subunit [EC:1.7.5.1 1.7.99.-]	ko00910; ko02020; ko01120; ko01100
	gene1549	Scaffold6	narI	K00374	nitrate reductase gamma subunit [EC:1.7.5.1 1.7.99.-]	ko01120; ko01100; ko02020; ko00910
	gene1550	Scaffold6	narJ	K00373	nitrate reductase molybdenum cofactor assembly chaperone NarJ/NarW	ko02020
	gene1553, gene1554	Scaffold6	narK	K02575	MFS transporter, NNP family, nitrate/nitrite transporter	ko00910
	gene0585	Scaffold2	nirB	K00362	nitrite reductase (NADH) large subunit [EC:1.7.1.15]	ko00910; ko01120; ko01100
**Ammonia assimilation**	gene0795, gene1018	Scaffold3, Scaffold 4	glnB	K04751	nitrogen regulatory protein P-II 1	ko02020
	gene2209	Scaffold10	glnD	K00990	[protein-PII] uridylyltransferase [EC:2.7.7.59]	ko02020

gene3699	Scaffold26	glnL	K07708	two-component system, NtrC family, nitrogen regulation sensor histidine kinase GlnL [EC:2.7.13.3]	ko02020
gene3698	Scaffold26	glnG	K07712	two-component system, NtrC family, nitrogen regulation response regulator GlnG	ko02020
gene1937	Scaffold8	glnA	K01915	glutamine synthetase [EC:6.3.1.2]	ko04727; ko04217; ko01120; ko00220; ko01100; ko02020; ko00250; ko00910; ko01230; ko00630; ko04724
gene3353	Scaffold21	-	K01501	nitrilase [EC:3.5.5.1]	ko01120; ko00643; ko00627; ko00380; ko01100; ko00910; ko00460
gene3133	Scaffold18	-	K15371	glutamate dehydrogenase [EC:1.4.1.2]	ko01120; ko00220; ko01100; ko00910; ko00430; ko00250
gene4137	Scaffold37	gdhA	K00262	glutamate dehydrogenase (NADP+) [EC:1.4.1.4]	ko00220; ko01120; ko01100; ko00910; ko00250
gene2064	Scaffold9	glsA	K01425	glutaminase [EC:3.5.1.2]	ko04727; ko04964; ko00470; ko00220; ko05230; ko01100; ko00250; ko05206; ko02020; ko04724
gene2085	Scaffold9	glmS	K00820	glutamine---fructose-6-phosphate transaminase (isomerizing) [EC:2.6.1.16]	ko00520; ko05415; ko00250; ko01100; ko04931; ko01250
gene3333	Scaffold21	carB	K01955	carbamoyl-phosphate synthase large subunit [EC:6.3.5.5]	ko00250; ko00240; ko01100; ko01240
gene1260	Scaffold5	purF	K00764	amidophosphoribosyltransferase [EC:2.4.2.14]	ko00230; ko01110; ko01100; ko00250

**Ammonifying function**	gene0061	Scaffold1	pepO	K07386	putative endopeptidase [EC:3.4.24.-]	
	gene0658, gene4010	Scaffold2, Scaffold32	ptrA	K01407	protease III [EC:3.4.24.55]	
	gene0847, gene0966, gene4341	Scaffold3, Scaffold4, Scaffold51	ina	K09607	immune inhibitor A [EC:3.4.24.-]	
	gene0854, gene0959, gene0855, gene0958, gene2830, gene0959, gene0855	Scaffold3, Scaffold4, Scaffold3, Scaffold4, Scaffold15, Scaffold4, Scaffold3	-	K23743	peptidyl-Asp metalloendopeptidase [EC:3.4.24.33]	
	gene2107, gene2620, gene2662, gene3259	Scaffold9, Scaffold13, Scaffold13, Scaffold20	lasA	K08642	LasA protease [EC:3.4.24.-]	ko02024
	gene2657, gene3295	Scaffold13, Scaffold20	colA	K01387	microbial collagenase [EC:3.4.24.3]	
	gene3193	Scaffold19	prlC	K01414	oligopeptidase A [EC:3.4.24.70]	
	gene3840	Scaffold29	pqqL	K07263	zinc protease [EC:3.4.24.-]	

	gene0474, gene0490, gene0863, gene0950, gene1361, gene2066, gene2356, gene3174, gene4005	Scaffold2, Scaffold2, Scaffold3, Scaffold4, Scaffold6, Scaffold9, Scaffold10, Scaffold19, Scaffold32	-	K14645	serine protease [EC:3.4.21.-]	ko02024
	gene0802, gene1011	Scaffold3, Scaffold4	lexA	K01356	repressor LexA [EC:3.4.21.88]	
	gene1243, gene1679	Scaffold5, Scaffold7	lon	K01338	ATP-dependent Lon protease [EC:3.4.21.53]	ko04112
	gene1287	Scaffold5	sppA	K04773	protease IV [EC:3.4.21.-]	
	gene1371, gene4051	Scaffold6, Scaffold33	pncB	K00763	nicotinate phosphoribosyltransferase [EC:6.3.4.21]	ko00760; ko01240; ko01100
	gene2520, gene2666	Scaffold12, Scaffold13	aprX	K17734	serine protease AprX [EC:3.4.21.-]	
	gene2749	Scaffold14	hhoB	K04691	serine protease DegS [EC:3.4.21.-]	
	gene2821	Scaffold15	tri	K08676	tricorn protease [EC:3.4.21.-]	
	gene3103	Scaffold18	sohB	K04774	serine protease SohB [EC:3.4.21.-]	
	gene3787	Scaffold28	degP	K04771	serine protease Do [EC:3.4.21.107]	
	gene4056	Scaffold34	ptrB	K01354	oligopeptidase B [EC:3.4.21.83]	ko05142; ko05143
**Phosphate solubilizing**						
	gene1581	Scaffold6	phoA	K01077	alkaline phosphatase [EC:3.1.3.1]	ko01240; ko01100; ko00730;

					ko02020; ko00790
gene0848, gene0965	Scaffold3, Scaffold 4	phoD	K01113	alkaline phosphatase D [EC:3.1.3.1]	ko02020; ko00790; ko01240; ko01100
gene2477, gene 3724	Scaffold12, Scaffold 26	phoB	K07657	two-component system, OmpR family, phosphate regulon response regulator PhoB	ko02020
gene0521, gene3725	Scaffold2, Scaffold 26	phoR	K07636	two-component system, OmpR family, phosphate regulon sensor histidine kinase PhoR [EC:2.7.13.3]	ko02020
gene1781, gene3727	Scaffold7, Scaffold 26	pstS	K02040	phosphate transport system substrate-binding protein	ko05152; ko02020; ko02010
gene3730	Scaffold26	pstB	K02036	phosphate transport system ATP-binding protein [EC:7.3.2.1]	ko02010
gene3475	Scaffold23	PiT	K03306	inorganic phosphate transporter, PiT family	-
gene0079	Scaffold1	gdh	K19813	glucose dehydrogenase [EC:1.1.5.9]	ko01110; ko01100; ko00030
gene0457	Scaffold2	pqqC	K06137	pyrroloquinoline-quinone synthase [EC:1.3.3.11]	-

Table S5. The possibly partial important genes and gene clusters of strain GXAS 306^T involved in the synthesis of various vitamins and auxin

Vitamins type	KEGG Name	KO ID	KO Description	Gene ID	Location
**Thiamine B$_1$**	thiC	K03147	phosphomethylpyrimidine synthase [EC:4.1.99.17]	gene1541	Scaffold6
	thiDE	K14153	thiamine-phosphate diphosphorylase [EC: 2.5.1.3]	gene1537	Scaffold6
	phoD	K01113	alkaline phosphatase [EC:3.1.3.1]	gene0848, gene0965	Scaffold3, Scaffold4
	rsgA	K06949	thiamine phosphate phosphatase [EC:3.6.1.- 3.1.3.100]	gene2114, gene2563	Scaffold9, Scaffold12
	thiL	K00946	thiamine-monophosphate kinase [EC:2.7.4.16]	gene3951	Scaffold31
**Biotin**	bioF	K00652	8-amino-7-oxononanoate synthase [EC:2.3.1.47]	gene3848	Scaffold29
	bioA	K00833	adenosylmethionine-8-amino-7-oxononanoate aminotransferase [EC:2.6.1.62]	gene2261	Scaffold10
	bioD	K01935	dethiobiotin synthetase [EC:6.3.3.3]	gene3845	Scaffold29
	bioB	K01012	biotin synthase [EC:2.8.1.6]	gene3849	Scaffold29
	birA	K03524	BirA family transcriptional regulator, biotin operon repressor / biotin- [acetyl-CoA-carboxylase] ligase [EC:6.3.4.15]	gene4201	Scaffold40
	bioC	K02169	malonyl-CoA O-methyltransferase [EC:2.1.1.197]	gene3846	Scaffold29
	bioH	K02170	pimeloyl-[acyl-carrier protein] methyl ester esterase [EC:3.1.1.85]	gene3847	Scaffold29
**Cobalamin B$_{12}$**	cobA	K19221	cob(I) alamin adenosyltransferase [EC:2.5.1.17]	gene2839	Scaffold15
	cobQ	K02232	adenosylcobyric acid synthase [EC:6.3.5.10]	gene2840	Scaffold15
	cobC	K02225	cobalamin biosynthesis protein CobC	gene2834	Scaffold15
	cobP	K02231	adenosylcobinamide kinase [EC:2.7.1.156] / adenosylcobinamide-phosphate guanylyltransferase [EC:2.7.7.62]	gene2842	Scaffold15
	cobS	K02233	adenosylcobinamide-GDP ribazoletransferase [EC:2.7.8.26]	gene2843	Scaffold15
	cobC	K02226	alpha-ribazole phosphatase [EC:3.1.3.73]	gene2841	Scaffold15
	cobU	K00768	nicotinate-nucleotide-dimethylbenzimidazole phosphoribosyltransferase [EC:2.4.2.21]	gene2844	Scaffold15
**Vitamin B$_6$**	pdxB	K03473	erythronate-4-phosphate dehydrogenase [EC:1.1.1.290]	gene0663	Scaffold2
	serC	K00831	phosphoserine aminotransferase [EC:2.6.1.52]	gene0202	Scaffold1
	pdxA	K00097	4-hydroxythreonine-4-phosphate dehydrogenase [EC:1.1.1.262]	gene1433	Scaffold6

	pdxJ	K03474	pyridoxine 5-phosphate synthase [EC:2.6.99.2]	gene1730	Scaffold7
	pdxH	K00275	pyridoxamine 5'-phosphate oxidase [EC:1.4.3.5]	gene3477	Scaffold23
**Riboflavin (Vitamin B_2)**	ribA	K01497	GTP cyclohydrolase II [EC:3.5.4.25]	gene1473	Scaffold6
	ribD	K11752	diaminohydroxyphosphoribosylaminopyrimidine deaminase [EC:3.5.4.26] / 5-amino-6-(5-phosphoribosylamino) uracil reductase [1.1.1.193]	gene3945	Scaffold31
	yigB	K20862	5-amino-6-(5-phospho-D-ribitylamino) uracil phosphatase [EC:3.1.3.104]	gene2632	Scaffold13
	ribBA	K14652	3,4-dihydroxy 2-butanone 4-phosphate synthase [EC:4.1.99.12]	gene3947	Scaffold31
	ribH	K00794	6,7-dimethyl-8-ribityllumazine synthase [EC:2.5.1.78]	gene3948	Scaffold31
	ribE	K00793	riboflavin synthase [EC:2.5.1.9]	gene3946	Scaffold31
	ribF	K11753	riboflavin kinase [EC:2.7.1.26] / FMN adenylyltransferase [EC:2.7.7.2]	gene0043	Scaffold1
**Indole**	trpA	K01695	tryptophan synthase alpha chain [EC:4.2.1.20]	gene3120	Scaffold18
	trpB	K01696	tryptophan synthase beta chain [EC:4.2.1.20]	gene3119	Scaffold18
	trpCF	K13498	indole-3-glycerol phosphate synthase [EC:4.1.1.48] / phosphoribosylanthranilate isomerase [EC:5.3.1.24]	gene3118	Scaffold18
	trpD	K00766	Anthranilate phosphoribosyltransferase [EC 2.4.2.18]	gene3117	Scaffold18
	-	K01501	nitrilase [EC:3.5.5.1]	gene3353	Scaffold21
**Polyamine**	speA	K01585	arginine decarboxylase [EC:4.1.1.19]	gene2275	Scaffold10
	aguA	K10536	agmatine deiminase [EC:3.5.3.12]	gene2274	Scaffold10
	aguB	K12251	N-carbamoylputrescine amidase [EC:3.5.1.53]	gene2273	Scaffold10
	speE	K00797	spermidine synthase [EC:2.5.1.16]	gene1534	Scaffold6
				gene1618	Scaffold7
				gene2276	Scaffold10
				gene4097	Scaffold36
	speD	K01611	S-adenosylmethionine decarboxylase [EC:4.1.1.50]	gene2277	Scaffold10
**Folate (Vitamin B_9)**	tilS	K04075	tRNA(Ile)-lysidine synthase [EC:6.3.4.19]	gene1782	Scaffold7
	hprT	K00760	hypoxanthine phosphoribosyltransferase [EC:2.4.2.8]	gene2910	Scaffold16
	ftsH	K03798	cell division protease FtsH [EC:3.4.24.-]	gene3478	Scaffold23
	folE	K01495	GTP cyclohydrolase IA [EC:3.5.4.16]	gene0660	Scaffold2
	folP	K00796	dihydropteroate synthase [EC:2.5.1.15]	gene3359	Scaffold21
	folB	K01633	7,8-dihydroneopterin aldolase [EC:4.1.2.25]	gene1420	Scaffold6

	folK	K00950	2-amino-4-hydroxy-6-hydroxymethyldihydropteridine diphosphokinase [EC:2.7.6.3]	gene1419, gene1476	Scaffold6
	panE	K00077	2-dehydropantoate 2-reductase [EC:1.1.1.169]	gene2222	Scaffold10
	panC	K01918	pantoate--beta-alanine ligase [EC:6.3.2.1]	gene1479	Scaffold6

Table S6. The possibly partial important genes and gene clusters of LysR-type transcriptional regulator (LTTR) of strain GXAS 306^{T}

KEGG Name	KO ID	KO Description	Gene ID	Location
cysB	K13634	cys regulon transcriptional activator	gene0087	Scaffold1
			gene1578	Scaffold6
			gene3075	Scaffold17
			gene2452	Scaffold11
nhaR	K03717	transcriptional activator of nhaA	gene2299	Scaffold10
oxyR	K04761	hydrogen peroxide-inducible genes activator	gene2476	Scaffold12
ilvY	K02521	positive regulator for ilvC	gene2701	Scaffold14
gcvA	K03566	glycine cleavage system transcriptional activator	gene2714<br>gene2763	Scaffold14
hdfR	K23773	flagellar master operon regulator	gene2930	Scaffold16
cbbR	K21703	low CO2-responsive transcriptional regulator	gene4156	Scaffold38
iciA	K05596	chromosome initiation inhibitor	gene4208	Scaffold40
ilvC	K00053	ketol-acid reductoisomerase [EC:1.1.1.86]	gene2702	Scaffold14
